# Mesenchymal stem cells in treating human diseases: molecular mechanisms and clinical studies

**DOI:** 10.1038/s41392-025-02313-9

**Published:** 2025-08-22

**Authors:** Xia Han, Rongdong Liao, Xiang Li, Cantong Zhang, Shaochuan Huo, Lei Qin, Yi Xiong, Tailin He, Guozhi Xiao, Tianfeng Zhang

**Affiliations:** 1 Research Institute, Shenzhen Hospital (Futian) of Guangzhou University of Chinese Medicine, Shenzhen, China; 2 Department of Orthopedics, Zhujiang Hospital, Southern Medical University, Guangzhou, China; 3 Guizhou Provincial Engineering Technology Research Center for Chemical Drug R&D, College of Pharmacy, Guizhou Medical University, Guiyang, China; 4 Department of Rheumatology and Immunology, Shenzhen Third People’s Hospital, Shenzhen, China; 5 Department of Orthopedics, Shenzhen Nanshan People’s Hospital, and the 6th Affiliated Hospital of Shenzhen University Medical School, Shenzhen, China; 6 CAS Key Laboratory of Pathogenic Microbiology and Immunology, Institute of Microbiology, Chinese Academy of Sciences (CAS), Beijing, China; 7 Department of Biochemistry, Homeostatic Medicine Institute, School of Medicine, Shenzhen Key Laboratory of Cell Microenvironment, Guangdong Provincial Key Laboratory of Cell Microenvironment and Disease Research, Southern University of Science and Technology, Shenzhen, China

**Keywords:** Stem-cell research, Molecular medicine

## Abstract

Mesenchymal stem cells (MSCs) have emerged as a highly promising strategy in regenerative medicine due to their self-renewal, pluripotency and immunomodulatory properties. MSCs are nonhematopoietic, multipotent stem cells that can differentiate into various mesodermal lineages and modulate the immune system. The therapeutic potential of MSCs from different tissues has been widely explored in preclinical models and clinical trials for human diseases, ranging from autoimmune diseases and inflammatory disorders to neurodegenerative diseases and orthopedic injuries. The therapeutic effects of MSCs can be mediated through the release of bioactive molecules, including growth factors, cytokines, and extracellular vesicles, which play crucial roles in modulating the local cellular environment, promoting tissue repair, angiogenesis, and cell survival, and exerting anti-inflammatory effects. MSCs can also interact with various immune cells, such as T cells, B cells, dendritic cells, and macrophages, modulating the immune response through both direct cell‒cell interactions and the release of immunoregulatory molecules. This review delves into the molecular mechanisms, signaling pathways, and regulatory factors that underpin the therapeutic effects of MSCs. This review also highlights the clinical applications and challenges associated with the use of MSC-based drugs to promote the safety and efficacy of MSC-based therapies. Overall, this comprehensive review provides valuable insights into the current state of MSC research and its potential for transforming the field of regenerative medicine as well as immune-mediated inflammatory diseases.

## Introduction

Stem cell therapy has emerged as a promising approach in regenerative medicine, offering the potential to repair or replace damaged tissues and organs.^1^ Among various types of stem cells, mesenchymal stem cells (MSCs) have garnered significant interest because of their unique biological properties, versatility, and clinical safety profile.^2^ Originally identified in the bone marrow, MSCs have since been isolated from a variety of tissues, including adipose tissue, umbilical cord blood, dental pulp, and placental tissue.^3^ These adult stem cells are characterized by their capacity for self-renewal, multilineage differentiation, and immunomodulatory functions, making them attractive candidates for therapeutic applications across a broad spectrum of human diseases.^4–6^

MSCs are nonhematopoietic, multipotent stem cells that can differentiate into various mesodermal lineages, including osteoblasts, chondrocytes, adipocytes, and ectodermal and endodermal lineages.^7^ The ability of stem cells to modulate the immune system and promote tissue repair distinguishes them from other stem cell types. According to the International Society for Cellular Therapy (ISCT, a nongovernmental organization), MSCs are defined on the basis of three key criteria: (1) adherence to plastic when cultured under standard conditions; (2) expression of specific surface markers (such as CD73, CD90, and CD105; ≥95%) while lacking expression of hematopoietic markers (CD34, CD45, CD14 or CD11b, CD79α or CD19, HLA-DR; ≤2%); and (3) the capacity to differentiate into osteogenic, chondrogenic, and adipogenic lineages under in vitro conditions.^8^ Among the positive markers, CD105 is a type I membrane glycoprotein essential for cell migration and angiogenesis.^9^ CD90, an N-glycosylated glycosylphosphatidylinositol, is part of the immunoglobulin superfamily and was initially identified for its role as a thymic cell antigen.^10^ It mediates cell‒cell and cell‒extracellular matrix (ECM) interactions, contributing to intercellular adhesion and migration.^11^ CD90 is broadly expressed on the membrane surfaces of various stem cells, including MSCs,^12^ liver stem cells,^13^ and keratinocytes.^14^ CD73 functions as a 5’-exonuclease, catalyzing the hydrolysis of adenosine monophosphate into individual nucleotides.^15^ It may play a role in cell signaling within the bone marrow, modulating cellular interactions, particularly during the normal development of lymphocytes.^16^ CD45 serves as a marker for white blood cells,^17^ whereas CD34 is a biomarker for hematopoietic stem cells and endothelial cells.^18^ CD14 and CD11b are expressed in monocytes and macrophages,^19^ whereas CD79α and CD19 are markers of B cells.^20^ HLA-DR is an MHC-II molecule found in B lymphocytes, monocytes, macrophages, activated T lymphocytes, and activated natural killer (NK) lymphocytes and exhibits strong immunogenic properties.^21^

MSCs can be classified into various types on the basis of their tissue of origin. Bone marrow-derived MSCs (BM-MSCs) are the most extensively studied type and are known for their high differentiation potential and strong immunomodulatory effects.^22^ Compared with BM-MSCs, adipose tissue-derived MSCs (AD-MSCs) are easier to harvest and yield more easily, with comparable therapeutic properties.^23^ Umbilical cord-derived MSCs (UC-MSCs) are known for their enhanced proliferation and lower immunogenicity, making them suitable for allogeneic transplantation.^24^ In addition, dental pulp stem cells (DP-SCs) and placenta-derived MSCs (P-MSCs) are emerging sources with unique regenerative properties and specific applications in dental and obstetric medicine.^25,26^ The behavior and therapeutic potential of MSCs are tightly regulated by intrinsic and extrinsic factors, including microenvironmental cues, epigenetic modifications, and cytokine signaling, where MSCs release a diverse range of bioactive molecules, including growth factors, cytokines, and extracellular vesicles (EVs). These components play crucial roles in modulating the local cellular environment; promoting tissue repair, angiogenesis, and cell survival; and exerting anti-inflammatory effects. MSCs have been shown to interact with various immune cells, such as T cells, B cells, dendritic cells (DCs), and macrophages, modulating the immune response through both direct cell‒cell interactions and the release of immunosuppressive molecules.

The therapeutic potential of MSCs has been widely explored in preclinical models and clinical trials for a variety of human diseases ranging from autoimmune diseases and inflammatory disorders to neurodegenerative diseases and orthopedic injuries. Furthermore, the clinical application of MSCs holds promise as an alternative to traditional therapies. This review will delve into the molecular mechanisms, signaling pathways, and regulatory factors underlying the therapeutic effects of MSCs while also highlighting key findings from recent clinical studies and addressing current challenges and future perspectives.

## Research history and milestones in the study of MSCs

### Early discoveries and characterization

The foundational concept of MSCs originated in the 1960s when the team led by Soviet scientist A. J. Friedenstein conducted groundbreaking research that revealed the capacity of bone marrow cell transplantation to differentiate into osteoblasts in vivo.^27^ Subsequent experiments led to the identification of progenitor cells for both osteoblasts and hematopoietic cells.^28^ In 1971, the existence of osteogenic precursor cells was further validated through animal studies.^29^ By 1974, Friedenstein and coworkers had isolated a fibroblast-like cell from bone marrow via adherent culture, which exhibited colony-forming units (CFU-F) that could differentiate into osteoblasts and facilitate the formation of hematopoietic clones.^30,31^ In 1987, it was established that this category of bone marrow stromal cells possessed differentiation potential; after 20–30 passages of proliferation, these cells could still generate bone tissue after being implanted into the peritoneum of rats, leading to the designation of bone marrow stromal stem cells.^32,33^ By 1990, these cells were confirmed to differentiate into both osteoblasts and chondrocytes.^34^ In 1987, Dr. Charbord^35^ reported that bone marrow stromal cells cultured in horse serum displayed notable differences in their uptake of serum proteins compared with that of bone marrow fibroblasts. This finding revealed that MSCs are not fibroblasts despite their similar morphology when cultured in vitro. In 1991, a research team at Queen’s University Belfast in Northern Ireland successfully cultured MSCs from Wharton’s jelly portion of the human umbilical cord via a tissue block culture technique.^36^ During this period, MSCs differentiate into fibroblast-like cells capable of being passaged up to the sixth passage, exhibiting the ability to secrete collagen and cytokeratin. In 1991, Dr. Arnold Caplan proposed that MSCs originate from the mesoderm and designated them mesenchymal stem cells, drawing upon earlier research findings.^37^ This nomenclature has been widely embraced in many subsequent studies. However, the depth of research and comprehension regarding MSCs is limited, and their significant immunosuppressive properties and cytokine secretion capabilities have not yet been acknowledged.

### Advancement in understanding MSC biology

In 1992, it was explicitly proposed that MSCs function as trophoblast cells, facilitating the differentiation of hematopoietic stem cells into granulocytes, macrophages, and megakaryocytes.^38,39^ This facilitation is mediated by growth factors secreted by MSCs, including colony stimulating factor 1 (CSF-1), granulocyte–macrophage (GM)-CSF, granulocyte (G)-CSF, interleukin (IL)-6, c-kit ligand, and IL-3.^39^ In 1992, J N Beresford and colleagues^40^ established that MSCs can differentiate into both adipocytes and osteoblasts. The presence of dexamethasone in the culture medium predominantly drives MSC differentiation toward adipocytes. However, when dexamethasone is combined with 1,25-dihydroxyvitamin D3 in coculture conditions, adipocyte differentiation is suppressed, whereas the potential for osteoblast differentiation is significantly increased. The in vitro ability of MSCs to differentiate into adipocytes, osteoblasts, and chondrocytes was first described in *Science* in 1999, thereby initiating extensive research into the differentiation potential of MSCs in humans and anticipating their substantial contributions to regenerative medicine.^41^ In 2000, a laboratory at the University of Chile isolated mononuclear cells from umbilical cord blood and purified them through adherent culture to obtain MSCs.^42^ MSCs were then isolated and cultured from various tissues (such as adipose tissue, amniotic membrane, gingiva, thymus, placenta, synovium, fetal blood, fetal liver, and fetal lungs) across different laboratories. In the same year, Dr. Charbord initiated efforts to identify MSCs via specific cell surface markers, including CD45, HLA-DR, CD33, CD11c, von Willebrand factor, alpha-smooth muscle actin, fibronectin, CD68, and CD51. In 1993, additional positive surface markers [such as CD10, CD44, CD29, VLA-1, VLA-5, and vascular cell adhesion molecule 1 (VCAM-1)] and negative markers (such as CD14, CD34, CD45, LFA-1a, LFA-1b, LFA-3, VLA-2, VLA-4, VLA-6, and Stro-1) associated with MSCs were further identified.^43^

By the mid-2000s, researchers had begun to uncover the mechanisms by which MSCs exert their therapeutic effects. MSCs can modulate immune responses by interacting with various immune cells, such as T cells, macrophages, and DCs.^44,45^ This immunomodulatory effect has become a key factor in the development of MSC-based treatments for rheumatoid arthritis, Crohn’s disease, and graft-versus-host disease (GVHD). Moreover, the concept of the “paracrine effect” has become widely accepted, where MSCs, through the secretion of bioactive factors, such as cytokines, growth factors, and extracellular vesicles, mediate tissue repair and immune modulation.^46,47^ These findings suggest that MSCs may function not only by differentiating into specific cell types or through cell‒cell interactions but also through the transfer of molecular signals via paracrine effects.

### Breakthroughs in MSC therapies and expanding clinical applications

Clinical investigations involving MSCs began to emerge in the 1990s. The first clinical study on MSCs was reported in 1995, in which adherent stromal cells were isolated and cultured from the bone marrow of patients suffering from malignant leukemia.^48^ These cells were subsequently reinfused into patients to evaluate their clinical efficacy and establish the safety profile of these stromal cells. This research represents a milestone in MSC research, facilitating the transition of MSC studies from laboratory settings to practical clinical applications.

In 2000, human bone marrow-derived MSCs were administered into the peritoneal cavity of sheep fetuses at gestational ages of 65 and 85 days (compared with 145 days of human gestation), and these cells were found to persist in various tissues for up to 13 months posttransplantation.^49^ The transplanted human bone marrow MSCs differentiated into chondrocytes, adipocytes, cardiomyocytes, bone marrow stromal cells, and thymic stromal cells in newborn sheep. This research unequivocally illustrates the multipotent differentiation potential of MSCs. The potent immunosuppressive properties of MSCs were established in 2002, revealing that even allogeneic or cross-species applications do not elicit immune rejection responses.^50–52^ In 2004, Blanc et al.^53^ published the first clinical study addressing the immunosuppressive effects of MSCs in the treatment of isolated GVHD, significantly advancing the clinical exploration of the use of MSCs for a variety of immune disorders. The discovery of the immunosuppressive capabilities of MSCs significantly advanced clinical studies for the treatment of various immune-mediated disorders.

In July 2011, the Ministry of Foods and Drug Safety of Korea granted approval for the commercialization of Hearticell-AMI, an MSC-based therapeutic developed by FCB Pharmicell for acute myocardial infarction in South Korea.^54^ Hearticellgram-AMI has been distinguished itself as the first autologous MSC therapy used globally for acute myocardial infarction, utilizing the patient’s own bone marrow MSCs administered through local coronary artery injection. In January 2012, two consecutive production licenses for MSC therapies were authorized. Cartistem, developed by Medi Post, is an MSC therapy designed to address degenerative arthritis and knee cartilage injuries by isolating and culturing MSCs from umbilical cord blood.^55^ The Anteogen Cuepistem is indicated for the treatment of complex Crohn’s disease complicated by anal fistula, utilizing MSCs isolated and cultured from the patient’s own adipose tissue.^56^ In the 1990s, Professor Caplan and his team established Osiris Treatment Company, which produced an MSC injection (Prochymal), conditionally approved in Canada in 2012 for treating acute refractory GVHD in pediatric patients despite the failure of the world’s inaugural phase III clinical trial for MSC therapies.^57^ The data from this clinical trial have yet to be compiled and published. However, several articles have commented on the situation, analyzed the reasons for the trial’s failure and discussed potential future directions for development.^58,59^ The inability of cryopreservation can be attributed to various factors, including the isolation and cultivation methodologies of MSCs, the quality of MSCs, population heterogeneity, and the health status of the donors. Nevertheless, this setback has inspired extensive research into MSCs themselves. Consequently, the United Kingdom and the European Union revised their treatment guidelines in 2012 and 2014, respectively, endorsing the use of MSCs as a third-line therapy for Grade 2–4 acute GVHD (aGVHD).^60,61^ Subsequently, Mesoblast (an Australian biotechnology corporation) acquired Prochymal from Osiris and rebranded it as Ryocil (codenamed remestemcel-L) to advance clinical trials and broaden its therapeutic indications, including those for colitis and acute respiratory distress syndrome (ARDS). In Japan, JCR Pharmaceuticals launched remestemcel-L, which received market authorization under the brand name Temcell; however, the U.S. Food and Drug Administration (FDA) has consistently denied the marketing application for remestemcel-L.

In July 2016, the results of the second phase III clinical trial involving MSCs worldwide were published in *Lancet*.^62^ This was a multicenter randomized controlled trial of Cx601 aimed at treating complex perianal fistulas in patients with Crohn’s disease. This investigation included 212 participants, 107 receiving AD-MSC treatment and 105 receiving a placebo. Notably, 50% of patients treated with AD-MSCs achieved combined remission, whereas 34% of patients in the placebo cohort achieved remission after 24 weeks. The efficacy of adipose MSC treatment was significantly superior to that of the placebo. Long-term efficacy data for the use of Cx601 in treating complex perianal fistulas in patients with Crohn’s disease were published in 2018.^63^ At week 52, the percentage of patients who achieved combined remission with Cx601 (56.3%) was markedly greater than that of the control group (38.6%) (*P* = 0.01), with clinical remission rates of 59.2% versus 41.6% (*P* = 0.013). The incidence of adverse events in the Cx601 group was 76.7%, whereas it was 72.5% in the control group. The outcomes of this phase III clinical trial demonstrated significant superiority over those of the control group, leading to the approval of Cx601 (Alofisel) for the treatment of complex perianal fistulas in patients with Crohn’s disease, which received approval in March 2018 within the European Union and in October 2021 in Japan. The positive outcomes of this study indicate that Alofisel represents an innovative therapeutic agent that fills significant clinical gaps. A follow-up clinical trial evaluating Alofisel for the treatment of 14 patients suffering from Crohn’s disease with perianal fistula was carried out between October 2018 and April 2021, yielding a healing rate of 57.1%.^64^ However, in October 2023, Takeda Corporation announced the findings of a randomized, placebo-controlled trial involving a substantial cohort of 568 patients with Crohn’s disease complicated by perianal fistula. This study revealed that the primary endpoint of combined remission was not met at the 24-week mark, indicating a lack of therapeutic efficacy. Consequently, allofenel was withdrawn from the market in December 2024.

The global COVID-19 pandemic represents one of the most significant challenges in recent years.^65^ There is an urgent need to identify effective prevention strategies and therapeutic interventions. Global initiatives have focused on the research and development of novel therapies and the exploration of various methodologies, with MSC-based therapies emerging as particularly promising. Early clinical trials investigated the potential of MSCs to treat COVID-19-related lung injuries and reduce the cytokine storm in severe cases.^66^ The immunomodulatory properties of MSCs have shown promise in reducing inflammation and accelerating recovery in patients with severe/critical symptoms.^67^

On December 18, 2024, there was significant news regarding the approval by the US FDA of the first MSC therapy for commercial use, which was grounded in findings from a multicenter, single-arm clinical trial.^68^ This decision represents a pivotal advancement in the application of innovative cell-based therapies aimed at addressing life-threatening conditions that severely affect patients, including pediatric populations. The product, known as Ryoncil (remestemcel-L from Mesoblast Corporation), is a type of allogeneic BM-MSC that has faced repeated scrutiny and has been denied regulatory approval by the U.S. FDA on multiple occasions. It is indicated for the treatment of steroid-resistant acute graft-versus-host disease (SR-aGVHD) in children aged 2 months and older. The FDA’s approval has invigorated the MSC industry, providing significant momentum.

**Fig. 1:**
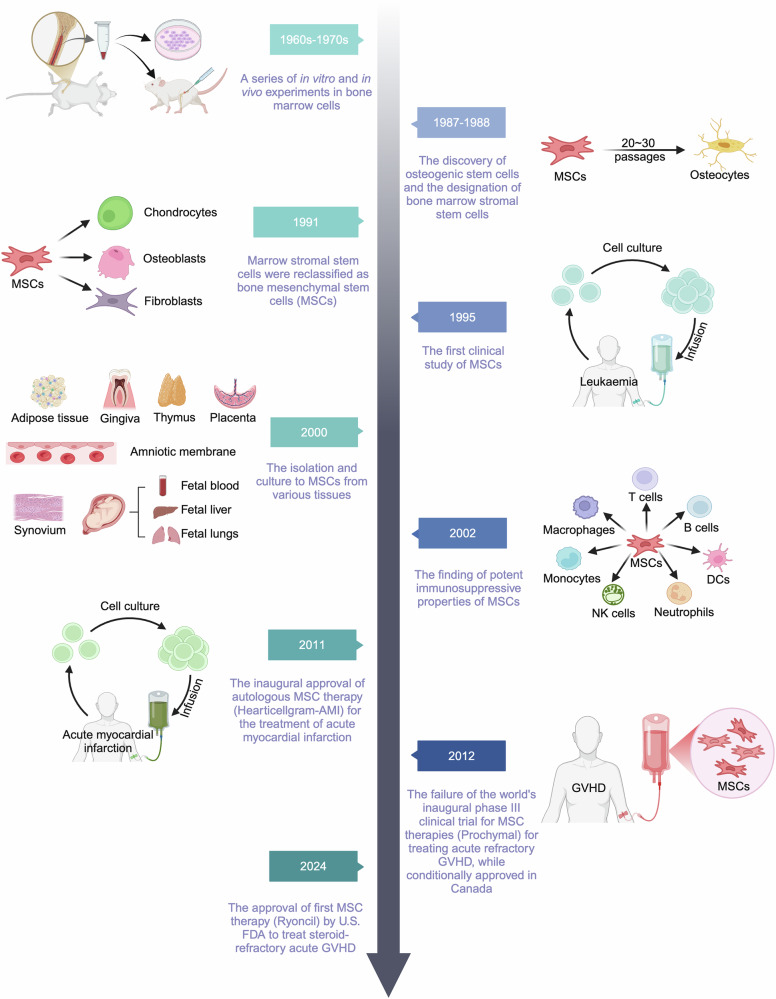
Research history and milestones in the study of MSCs and related treatments. The historical exploration of MSCs can be traced back to the 1960s, when bone marrow cell transplantation was first reported to facilitate in vivo differentiation into osteoblasts. A series of animal studies subsequently elucidated the multipotent differentiation capabilities of MSCs, their role in facilitating hematopoiesis, their diverse sources, their ability to modulate immune responses, and the characterization of their cell surface markers. Clinical investigations involving MSCs began to proliferate globally in the 1990s, aimed at addressing various human diseases, albeit accompanied by numerous challenges. As of the end of 2024, the U.S. Food and Drug Administration granted approval for the first MSC-based therapeutics for the treatment of SR-aGVHD in pediatric patients aged two months and older. The figure was created with BioRender.com. GVHD graft-versus-host disease, FDA Food and Drug Administration

The history of MSC research spans over six decades, beginning with their theoretical identification and culminating in clinical applications (Fig. 1). Today, MSCs are at the forefront of regenerative medicine, offering potential treatments for a wide range of diseases (Tables 1, 2). The field continues to evolve, with ongoing research addressing the optimization of MSC sources, improving differentiation techniques, and developing new applications for complex diseases. The future of MSC therapies will likely be shaped by advances in genetic engineering, biomaterials, and personalized medicine, making this an exciting and rapidly evolving field.

**Table 1:** MSC-therapeutic products for the treatment of various diseases

MSC product name	Company	Approved Country	Year	Indication	Cell source	Route	Frequency	Dose
Hearticellgram-AMI	Pharmicell Co. Ltd.	South Korea	2011	Acute myocardial infarction	Autologous BM	Coronary arterial injection	Single injection; Readministration may be considered	weight 60 kg or less: 10 ml/5 × 10^7^ cells; 61–80 kg: 14 ml/7 × 10^7^ cells; 81 kg or more: 18 ml/9 × 10^9^ cells
Cartistem	Medipost Co. Ltd.	South Korea	2012	Knee articular cartilage defects	Allogeneic UC	Local administration (surgical area)	Single injection; Readministration may be considered	2.5 × 10^6^ cells/500 μL/cm^2^ (size of knee cartilage defects)
Cupistem	Anterogen Co. Ltd.	South Korea	2012	Crohn’s fistula	Autologous BM	Local administration (surgical area)	Single injection; Readministration may be considered	1 × 10^7^ cells/cm^2^ of the fistula surface area
Prochymal	Osiris Therapeutics Inc., Mesoblast Ltd.	Canada	2012	GVHD	Allogeneic BM	i.v.	Twice, weekly	10^8^ unit/15 mL
Neuronata-R	Corestem Inc.	South Korea	2014	Amyotrophic lateral sclerosis	Autologous BM	Intrathecal injection	Twice, monthly	4.0 × 10^7^ cells/4 mL
Temcell HS	JCR Pharmaceuticals	Japan	2015	GVHD	Allogeneic BM	i.v.	Twice a week for 4 weeks	2 × 10^6^ cells/kg body weight/18 mL
Stempeucel	Stempeutics Research PVT	India	2016	OA	Allogeneic BM	Intra-articular injection	Single injection; Readministration may be considered	25–150 million cells
Stempeucel	Stempeutics Research PVT	India	2016	Critical limb ischemia	Allogeneic BM	Intramuscular injection	Single injection; Readministration may be considered	200–400 million cells
Alofisel	TiGenix NV/Takeda	European Union	2018	Complex perianal fistulas in Crohn’s disease	Allogeneic Adipose	50% into the fistula walls, and the remaining into the surrounding tissue	Single injection; Readministration may be considered	120 million cells
Stemirac	Nipro Corp	Japan	2018	Spinal cord injury	Autologous BM	i.v.	Single injection; Readministration may be considered	50–200 million cells within 3–8 weeks after the injury
Mesestrocell	Cell Tech Pharmed	Iran	2018	OA	Allogeneic BM	Intra-articular injection	Single injection	2 × 10^7^ cells/knee
Vartocell	Cell Tech Pharmed	Iran	2020	Spastic cerebral palsy	Allogeneic UC	Intrathecal and intracoronary injection	N.A.	2 × 10^7^ cells
Akuugo	SanBio Co., Ltd.	Japan	2024	Chronic motor paralysis caused by traumatic brain injury	Allogeneic BM	Intralesional injection	Single injection	5 × 10^6^ cells
Ryoncil	Mesoblast	USA	2024	SR-aGVHD	Allogeneic BM	i.v.	Twice a week for 4 consecutive weeks	2 × 10^6^ cells/kg
Aimmexitocel	Platinum Life	China	2025	SR-aGVHD	Allogeneic UC	i.v.	Twice a week for 4 or 8 consecutive weeks	1 × 10^6^ cells/kg

*i.v.* intravenous, *BM* bone marrow, *UC* umbilical cord, *GVHD* graft-versus-host disease, *OA* osteoarthritis, *SR-aGVHD* steroid-resistant acute graft-versus-host disease, *N.A.* not applicable

**Table 2:** Recent advancements in clinical trials utilizing MSCs for the treatment of diverse diseases

Diseases	Cell source	Route	Dose	Frequency	Patients (experiment group)	Clinical Trials number	Study phase	Outcomes	Ref
GVHD	BM	i.v.	2 × 10^6^ cells/kg	Twice a week, in total four weeks	54	NCT02336230	III	Remestemcel-L infusions significantly improved overall response in pediatric patients with SR-aGVHD and were well tolerated with no identified safety concerns. The improved response on day 28 was strongly associated with significantly improved survival through day 180.	^68^
GVHD	BM	i.v.	2 × 10^6^ cells/kg	One, two, or four times a week	15	NCT02359929	I	No dose-limiting toxicities. Three patients had responses seen at any timepoint. No difference was seen among dose levels in peripheral blood lymphocyte subsets.	^470^
GVHD	BM	i.v.	1 × 10^6^ cells/kg	Once a week, 4 weeks	101	NCT02241018	III	The most common adverse events were infections and hematological toxicity. The OR on day 28 and 56 was higher in the MSC group. The median failure-free survival was also longer in the BM-MSCs group. The cumulative incidence of cGVHD was lower than the control group.	^471^
GVHD	BM	i.v.	2 × 10^6^ cells/kg	At least 6 times	11	NCT01522716	II	Displayed a distinct immune phenotype characterized by higher levels of naive T cells and B cells, and a significantly higher fraction of CD31+ naive CD4 + T cells.	^472^
GVHD	BM	i.v.	1.5–3 × 10^6^ cells/kg	Once	23	NCT01045382	NA	One-year OS remain no change. No difference was observed in the incidences of chronic GVHD, infection or relapse, overall or progression-free survival	^473^
GVHD	BM	i.v.	1.5 × 10^7^ cells/kg	Once	5	000024291	I	All patients demonstrated successful recovery of neutrophil, reticulocyte, and platelet. At 1 year, all MSC‐CBT patients survived without relapse.	^474^
GVHD	UC	i.v.	1 × 10^6^ cells/kg	Once	25	ChiCTR-INR-16008399	II	Promoted platelet engraftment and decreased severe acute GVHD without increasing relapse rate.	^475^
GVHD	UC	i.v.	1 × 10^6^ cells/kg	Once every 2 weeks, 4 doses in total	78	ChiCTR-IIR-16007806	NA	The MSC group had better GVHD-free and relapse-free survival rates than the control group.	^476^
GVHD	UC	i.v.	1–2 × 10^6^ cells/kg	Twice a week, 2–4 weeks in total	7	UMIN000032819	I	Three subjects showed complete response. NK cell counts significantly increased, whereas IL-12, IL-17, and IL-33 levels decreased.	^477^
GVHD	BM	i.v.	0.9–1.3 × 10^6^ cells/kg	Once	43	NCT01941394	NA	It exerted a positive effect on the restoration of T-cell subpopulations and immune system recovery.	^478^
GVHD	Extraembryonic fetal tissues	i.v.	2–10 × 10^6^ cells/kg	On day 0 and 7	10	NCT03158896	I	The ORR of clinical response on day 28 was 70%. Day 100 and 180 post infusion survival was 90% and 60%. Serum biomarker REG3α decrease correlated with clinical response.	^479^
GVHD	Induced pluripotent stem cells	i.v.	1 × 10^6^ or 2 × 10^6^ cells/kg	On day 0 and 7	16	NCT02923375	I	No serious adverse events. OR, CR and OS rates by day 100 were 86.7, 53.3 and 86.7%.	^480^
GVHD	BM	i.v.	1.0 × 10^6^ cells/kg	Once every 2 weeks, 4 doses in total	10	KCT0001894	I/II	It revealed a reduction in inflammatory markers. All patients showed amelioration of clinical symptoms and enhancement of their quality of life.	^481^
GVHD	UC	i.v.	1.0 × 10^6^ cells/kg	Twice a week, 4–8 weeks	40	ChiCTR2000035740	II	The 2-year cumulative incidence of moderate to severe cGVHD was marginally lower in MSC group than in the control.	^482^
GVHD	UC	i.v.	1.0 × 10^6^ cells/kg	A median of four times	86	NCT01754454	NA	24 patients achieved complete remission, while 21 exhibited partial remission. The survival rate was approximately 11.6%.	^483^
SLE	BM	i.v.	2–3 × 10^6^ cells/kg	Once	7	NCT03174587	I	3 adverse events were reported, one diarrhea, one toothache, and one arthralgia.	^484^
SLE	BM or UC	i.v.	1 × 10^6^ cells/kg	Once or twice	69	NCT00698191 andNCT01741857	NA	40 participants achieved low disease activity and 16 participants reached clinical remission.	^485^
SLE	UC	i.v.	1 × 10^6^ cells/kg	Once	6	NCT03171194	I	5 participants achieved the clinical endpoint. The autoantibody levels were decreased. Tregs of 2 participants increased, but other T cells had no significant changes.	^486^
SLE	Adipose	i.v.	2 × 10^6^ cells/kg	Once	9	NA	I	Allogenic AD-MSC transplantation was associated with favorable safety and efficient to reduce urine protein excretion and disease activity. Meanwhile, the greatest improvement happened at a month based on the proteinuria.	^487^
RA	BM	i.v.	1 × 10^6^ cells/kg	Once	13	NA	NA	Increased FOXP3, IL-10 and TGF-β1 in PBMCs.	^488^
RA	Adipose	i.v.	2 × 10^8^ cells	Once	15	NCT03691909	I/IIa	No adverse events. ACR66/68 scores significant improved, levels of TNF-α, IL-6 and ESR remained unchanged.	^489^
RA	UC	Intravenous drip	2 × 10^7^ cells	Once	64	NCT01547091	NA	The ESR, CPR, RF and anti-CCP after treatment were detected to be lower than that of pretreatment.	^490^
RA	UC	Intravenous drip	1 × 10^6^ cells/kg	Once	63	ChiCTR-INR-17012462	I/II	No unexpected safety issues, the efficacy and ACR20 response rates were higher.	^491^
UC	BM	i.v.	1.5 × 10^6^/kg	Once	4	NCT04543994	I/II	At 3 months, all patients were satisfied with MSC treatment based on the inflammatory bowel disease patient-reported treatment impact.	^492^
UC	UC	i.v.	1 × 10^8^ cells	Biweekly, 3 times in total	6	NCT03299413	I	No significant short-term or intermediate-term adverse events were detected post-UCMSC administration. Long-term follow-up at 12 and 24 months showed sustained safety and no adverse events.	^493^
CD	BM	Intralesional	3 × 10^7^ cells	Once	16	N.A.	N.A.	Open-label injection of BM-derived MSCs was safe and was effective in half of patients in fistulizing perianal CD and induced significant MRI changes associated with favorable clinical outcome.	^494^
CD	BM	Intralesional	3 × 10^7^ cells	Once	10	N.A.	I/II	4 patients were hospitalized due to occlusion. At 12 weeks, 5 patients presented a complete or partial resolution of the stricture. There were no adverse or serious adverse events related to MSC therapy. At 6 months, 31% of the treatment group and 20% of the control had complete clinical and radiographic healing.	^495^
CD	BM	i.v.	7.5 ×10^7^ cells	Once or twice	16	NCT04519684	I/II	No adverse events. PCDAI, Wexner incontinence score, and van assche score significantly decreased in treatment patients.	^496^
CD	BM	Intralesional	1–9 × 10^7^ cells	Once	21	NCT01144962	N.A.	Two malignancies were observed. No serious adverse events. 63%, 100%, and 43% of the fistulas were closed in 3 cohorts, respectively. MRI showed significantly smaller fistula tracts.	^141^
CD	BM	Intralesional	7.5 × 10^7^ cells	Once	10	CTRI/2020/01/022743	I/II	At week 52, two patients were in remission and seven maintained responses. At 104 weeks, two patients maintained response and one was in remission.	^497^
CD	BM	Intralesional	7.5 × 10^7^ cells	Once	7	N.A.	I	No adverse events. At 6 months, 83% had complete clinical and radiographic healing.	^498^
CD	BM	Intralesional	7.5 × 10^7^ cells	Once	15	N.A.	Ib/IIa	50% of the treatment group had complete clinical and radiographic healing; 91.7% of the treatment group had improvement	^499^
CD	Adipose	Intralesional	N.A.	Once	20	N.A.	I	4 patients experienced 7 serious adverse events and 12 patients experienced 22 adverse events.	^500^
CD	Adipose	Intralesional	1.2 × 10^8^ cells	Once	25	NCT01541579	III	7 serious adverse events occurred. Clinical remission was reported in 56% patients.	^501^
CD	Adipose	Intralesional (MSCs-Matrix)	N.A.	Once	5	N.A.	I	All patients had a reduction in the size of fistula tract, and 3 of 5 had cessation of drainage, but none achieved complete healing.	^502^
CD	UC	Intralesional	1.2 × 10^8^ cells	Once	10	NCT04939337	N.A.	No adverse events occurred. The fistulas decreased, Significant improvement in Perianal CD Activity Index, Pelvic MRI-Based Score, and quality of life score on 24 W.	^503^
CD	Adipose	Intralesional	1.09–4.78 × 10^6^ cells/kg	Once	10	N.A.	N.A.	7 patients had clinical responses. A decreased PDAI score and an increased quality of life score.	^504^
CD	Adipose	Intralesional	2 × 10^7^ cells	Once	15	N.A.	I	No serious adverse events. 3 patients had complete clinical healing, 8 patients had partial healing, and 4 patients showed no clinical improvement.	^505^
CD	Adipose	Intralesional	N.A.	One, two or three times	21	NCT03803917	I	57% of patients had complete fistula healing.	^506^
CD	UC	i.v.	1 × 10^6^ cells/kg	8 doses in 5 months	8	N.A.	I	The clinical symptoms and signs of patients with CD were substantially relieved (CDAI, CRP, ESR). The genus *Cetobacterium* was significantly enriched	^507^
CD	BM	Intralesional	1.5–3 × 10^8^ cells	Once	6	NCT04548583	Ib/IIa	Seven adverse events occurred. SES-CD and CDAI score decreased.	^508^
ARDS	BM	i.v.	1 × 10^7^ cells/kg	Once	60	NCT02097641	II	No patient experienced any adverse events. 28-day mortality did not differ between the groups, The MSC group had higher APACHE III scores, minute ventilation and PEEP.	^509^
COVID-19	BM-MSC-EV	i.v.	15 mL	Once	25	NCT04493242	N.A.	The treated patients had a restored oxygenation, reduced cytokine storm and modulated immunity.	^510^
COVID-19 ARDS	BM	i.v.	1 × 10^6^ cells/kg	2 or 3 doses	23	432/2020BO	I	The MSCs infusion was safe. The group had a significantly higher Horovitz score on discharge.	^511^
COVID-19 ARDS	BM	i.v.	1 × 10^6^ cells/kg	Once	20	NCT04615429	N.A.	It was safe and may be beneficial, in low mortality, accelerating clinical recovery and hospital discharge	^512^
COVID-19 ARDS	UC	i.v.	1 × 10^8^ cells	Once or twice	19	IRCT20200217046526N2	II	It reduced the levels of inflammatory markers in patients, with no serious adverse events.	^513^
COVID-19 ARDS	Placenta	i.v.	1 × 10^6^ cells/kg	Once	10	IRCT20200621047859N4	I	No adverse events. Length of hospitalization, serum oxygen saturation, and other clinical and laboratory parameters were same.	^514^
COVID-19 ARDS	UC	i.v.	1 × 10^6^ cells/kg	3 times every other day	10	IRCT20160809029275N1	I	No serious adverse effects. It declined cytokine storm and recover respiratory functions	^515^
COVID-19 ARDS	BM	i.v.	9 × 10^8^ cells	Once	20	NCT03807804	II	No significant difference in the number of VFDs but may result in improved survival	^516^
COVID-19 ARDS	UC	i.v.	1 × 10^6^ cells/kg	3 times at 48 h intervals	22	NCT04333368	N.A.	No patient required oxygen support. Quality of life improved throughout the follow-up period, and the six-minute walking distance remained slightly impaired. No adverse effects	^517^
COVID-19 ARDS	Placenta	i.v.	1 × 10^6^ cells/kg	Once or twice	10	IRCT2017010531786N1	N.A.	It improved oxygenation and pulmonary infiltrates, decreased inflammatory cytokine level.	^518^
COVID-19 ARDS	BM	i.v.	1 × 10^7^ cells/kg	Once	17	NCT02097641	IIa	It significantly reduced airspace total protein, Ang-2, IL-6.	^519^
COVID-19 ARDS	BM	i.v.	2 × 10^6^ cells/kg	Twice	113	NCT04371393	N.A.	No adverse events. No differences in days alive off ventilation	^520^
COVID-19 ARDS	UC	i.v.	1 × 10^6^ cells/kg	Once	20	NCT04457609	N.A.	It increased the survival rate. The length of stay in the intensive care unit and ventilator usage were not significant, and no adverse events.	^521^
COVID-19 ARDS	UC	i.v.	2 × 10^8^ cells	3 times every other day	11	N.A.	N.A.	It improved respiratory distress and reduced inflammatory biomarkers (IL-6, IFN-γ, TNF-α).	^522^
COVID-19 ARDS	UC	i.v.	0.8–1.2 × 10^8^ cells	Twice at days 0 and 3	12	N.A.	I/IIa	No serious adverse events. Inflammatory cytokines were decreased. patient survival and time to recovery were improved.	^523^
COVID-19 ARDS	UC	i.v.	1–10 × 10^6^ cells/kg	Once	9	1066023736	I	No adverse events. It decreased inflammatory biomarkers and increased immune cell markers	^524^
COVID-19	BM	i.v.	1 × 10^6^ cells/kg	Once	7	ChiCTR2000029990	N.A.	The pulmonary function and symptoms of all patients were significantly improved. 2 common and 1 severe patient were recovered. Peripheral lymphocytes level increased. CRP decreased. The level of TNF-α decreased, while the level of IL-10 increased.	^525^
COVID-19	BM	i.v.	1.5–3 × 10^6^ cells/kg	Every 3 days, 3 doses in total	8	NCT04445454	I/II	The BMSCs group shows very promising efficacy in severe COVID-19, with a higher survival compared to control patients.	^526^
COVID-19	BM-MSC-EV	i.v.	10 mL or 15 mL	Once or twice	102	NCT04493242	II	No treatment-related adverse events. Mortality (60-day) in the intention-to-treat population was reduced. Ventilation-free days improved for all patients.	^527^
COVID-19	Placenta	i.v.	1.5–2 × 10^9^ EVs/kg	Twice in two days	21	IRCT20130812014333N164	N.A.	hPMSC-sEVs reduced the mortality rate in the intervention group compared to the controls.	^528^
OI	BM	i.v.	4 × 10^6^ cells/kg	Once every 5–6 months, 5 times in total	2	NCT02172885	I	Elicited a pro-osteogenic paracrine response in patients and improved the qualities of life by bettering bone parameters.	^529^
OI	Fetal liver	i.v.	3 × 10^6^ cells/kg	Once every month for 4 months	18	NCT03706482	I/II	Not yet public	^530^
OA	UC	Intra-articular	2 × 10^7^ cells	Once	10	NCT06078059	N.A.	Not yet public	^531^
OA	UC	Intra-articular	2 × 10^7^ cells	On 0 and 6th month	9	NCT02580695	I/II	Pain visual analog scale and WOMAC score decreased significantly	^532^
OA	BM	Intra-articular	2.5 × 10^7^ cells	Once	65	CTRI/2018/09/015785	III	WOMAC and visual analog scale scores were significantly improved, the cartilage volume remained unchanged, and there was no deterioration observed in the deep cartilage of the medial femorotibial compartment of the knee	^533^
OA	Adipose	Intra-articular	1 × 10^8^ cells	Once	18	IRCT20080728001031N23	II	Decreased hyaluronic acid and cartilage oligomeric matrix protein levels in blood serum in patients, and affected multiple biomarkers in blood samples.	^534^
OA	UC	Intra-articular	1 × 10^7^ units	Every week for 3 weeks	29	NCT03800810	II	hUC-MSCs decreased pain in severe KOA and improved the knee functions in combination with HA.	^535^
OA	BM	Intra-articular	1–50 × 10^6^ cells	Once	12	NCT02351011	I/II	There were significant improvements in KOOS pain, symptoms, and quality of life and WOMAC stiffness, with the most obvious improvements in the 50 million dose cohort	^536^
OA	Adipose	Intra-articular	1 × 10^8^ cells	Once	12	N.A.	IIb	Functional improvement and pain relief without causing apparent adverse events at 6 months’ follow-up.	^537^
OA	Adipose	Intra-articular	1 × 10^8^ cells	Once	3	IRCT20080728001031N23	I	VAS, WOMAC and KOOS were significantly improved, with no serious adverse events	^538^
SCI	BM and UC	Intrathecal	1 × 10^6^ cells/kg	Biweekly, 3 times in total	20	NCT04288934	I/II	MSCs enhanced recovery from spinal cord injury and facilitate motor function improvements	^539^
Stroke	BM	Intranasal	4.5–5 × 10^7^ cells	Once	10	NCT03356821	N.A.	Safe and feasible.	^540^
Stroke	Adipose	i.v.	1 × 10^6^ cells/kg	Once	15	NCT04280003	IIb	Not yet public	^541^
Stroke	Adipose	i.v.	1 × 10^6^ cells/kg	Once	4	NCT01678534	IIa	Safe and no injection-related tumor developments were reported.	^542^
Stroke	BM	Intrathecal	1 × 10^6^ cells/kg	once every week for a month	59	ChiCTR-INR-16008908	II	Not yet public	^543^
Stroke	BM	i.v.	2 × 10^6^ cells/kg	Once	9	NCT01461720	II	The absolute change of median infarct volume in patients treated with MSCs was improved, and the average cumulative number of GEL was decreased.	^544^
MI	UC	Intracoronary	2 × 10^7^ cells	Once	20	IRCT20201116049408N1	N.A.	Not yet public	^545^
MI	UC	Intracoronary	1 × 10^7^ cells	Once	130	NCT05043610	III	Not yet public	^546^
MS	UC	i.v. and intrathecal	3 × 10^8^ or 1.5 × 10^7^ cells	1 or 2 doses	35	NCT03326505	I/II	Both groups showed better motor function, radiology, cognitive, sleep, immunological outcomes. Two doses can be more beneficial.	^547^
AD	UC	Lateral ventricle	1 or 3 × 10^7^ cells	Once every 3 months, 4 times in total	9	NCT02054208, NCT03172117	I	Total tau, phosphorylated tau, and Aβ in the CSF samples were reduced. Galectin-3, sICAM-1, progranulin and GDF-15 were increased one day after MSC transplantation, while decreased sharply within 4 weeks.	^548^
ALS	BM	Intrathecal	1 × 10^6^ cells/kg	Once	24	NCT0291768	I/II	Identified 220 deregulated proteins in CSF of ALS patients treated with MSCs by Proteomics analysis.	^549^
ALS	BM	Intrathecal	1 × 10^6^ cells/kg	2 or 5 doses	69	NCT04745299, KCT0005954	III	Not yet public	^550^
ALS	BM	Intrathecal and Intramuscular	1.25 × 10^8^ cells and 4.8 × 10^7^ cells	Once	36	NCT02017912	II	Rate of disease progression was improved at early time points. CSF neurotrophic factors increased and CSF inflammatory biomarkers decreased.	^551^
Diabetes	BM	i.v.	1 × 10^6^ cells/kg	Twice, on week 0 and 3	11	NCT04078308	I/II	Early autologous MSC transplant in new T1D children with hypoglycemia is safe and promising.	^412^
Diabetes	UC	i.v.	2.5–20 × 10^7^ cells	Once	14	NCT03406585	I/II	ProTrans MSCs are safe for new-onset T1D and may preserve beta cells.	^552^
Diabetes	UC	i.v.	1 × 10^6^ cells/kg	3 times in 4-week intervals	37	NCT02302599	N.A.	Men with T2DM and high AUCC-pep are more likely to benefit from UC-MSC treatment.	^553^
Diabetes	UC	i.v.	1 × 10^6^ cells/kg	Once a week for 3 weeks	24	ChiCTR2200057370	II	hUC-MSCs are safe for T2DM, causing mild side effects, and may modulate immunity and lymphocytes.	^554^
Diabetes	UC	i.v.	1 × 10^6^ cells/kg	3 times at 4-week intervals	45	NCT02302599	II	The MSCs group reduced insulin percentage, HbA1c level and increased the glucose infusion rate compared with the placebo group.	^415^
Diabetes	UC	i.v.	1 × 10^6^ cells/kg	Twice in 3 months	27	ChiCTR2100045434	N.A.	It is safe for new-onset T1D patients and may preserve islet β cells in the initial year following diagnosis better than standard care alone.	^555^
Diabetes	UC	i.v.	2 × 10^5^ cells/kg	3 times on 0/7/28 day	14	ChiCTR2200055885	I	hUC-MSCs benefit DFU healing, with good tolerance, faster healing, and a higher 3-year amputation-free survival rate.	^556^
Prostate Cancer	BM	i.v.	1 × 10^6^ or 2 × 10^6^ cells/kg	Once	7	NCT01983709	I	Unmodified MSCs do not seem viable for delivering antitumor therapy to primary prostate cancer.	^557^

*i.v.* intravenous, *GVHD* graft-versus-host disease, *SLE* Systemic lupus erythematosus, *RA* Rheumatoid arthritis, *UC* ulcerative colitis, *CD* Crohn’s disease, *ARDS* Acute respiratory distress syndrome, *COVID-19* Coronavirus disease 2019, *OI* Osteogenesis imperfecta, *OA* Osteoarthritis, *SCI* spinal cord injury, *MI* myocardial infarction, *MS* multiple sclerosis, *AD* Alzheimer’s disease, *ALS* amyotrophic lateral sclerosis, *BM* bone marrow, *UC* umbilical cord, *BM-MSC-EV* bone marrow mesenchymal stem cell-derived extracellular vesicles, *N.A.* not applicable, *OR* overall response, *OS* overall survival, *CBT* cognitive behavioral therapy, *IL-17* overall survival, *IL-33* interleukin-33, *ORR* overall response rate, *REG3a* regenerating islet-derived protein 3a, *OR* odds ratio, *CR* complete response, *FOXP3* forkhead box P3, *IL-10* interleukin-10, *TGF-β1* transforming growth factor-beta 1, *PBMCs* peripheral blood mononuclear cells, *TNF-α* tumor necrosis factor-alpha, *ESR* erythrocyte sedimentation rate, *CRP* C-reactive protein, *RF* rheumatoid factor, *anti-CCP* anti-cyclic citrullinated peptide, *HAQ* health assessment questionnaire, *DAS28* disease activity score 28, *MRI* magnetic resonance imaging, *CD* Crohn’s disease, *PDAI* Pouchitis disease activity index, *CDAI* Crohn’s disease activity index, *SES-CD* simple endoscopic score for Crohn’s disease, *APACHE III* Acute Physiology and Chronic Health Evaluation III, *PEEP* positive end-expiratory pressure, *VFDs* ventilator-free days, *PaO2/FiO2* partial pressure of arterial oxygen to fractional inspired oxygen, *Ang-2* angiopoietin-2, *IFN-γ* interferon-gamma, *WOMAC* Western Ontario and McMaster Universities Osteoarthritis Index, *KOA* knee osteoarthritis, *HA* hyaluronic acid, *KOOS* knee injury and osteoarthritis outcome score, *VAS* visual analog scale, *Aβ42* amyloid-beta 42, *CSF* cerebrospinal fluid, *CAM-1* cell adhesion molecule-1, *GDF-15* growth differentiation factor-15, *ALS* amyotrophic lateral sclerosis, *T1D* type 1 diabetes, *T2DM* type 2 diabetes mellitus, *AUCC* area under the curve of continuous data, *HbA1c* hemoglobin A1c, *DFU* diabetic foot ulcer

## MSCs and immune-mediated inflammatory diseases

### Graft-versus-host disease

GVHD is a serious, potentially deadly complication that arises following allogeneic hematopoietic stem cell transplantation. This condition occurs when T lymphocytes from the donor attack the recipient’s organs. GVHD symptoms are similar to those of autoimmune diseases and other immune disorders, primarily affecting the gastrointestinal tract, liver, lungs, and skin.^69^ GVHD can be classified into two types on the basis of the time of occurrence: aGVHD, which occurs within 100 days after transplantation, and chronic GVHD (cGVHD), which occurs 100 days after transplantation. Compared with aGVHD, cGVHD involves a wider range of organs, including the skin, gastrointestinal tract, liver, subcutaneous tissue, hair, lungs, oral cavity, esophagus, musculoskeletal system, and joints. The manifestations of cGVHD are more diverse and characteristic, often leading to fibrosis.^69^ Glucocorticoids, immunosuppressants, and other drugs are commonly employed in clinical settings to manage GVHD.^70^ However, prolonged usage of these medications can lead to drug resistance and adverse effects, thereby increasing the chances of transplant failure or infection. Consequently, novel approaches that offer superior safety and efficacy are urgently needed. Given the critical function of MSCs in modulating immune responses, their application in cell-based therapies for managing GVHD presents substantial promise, as evidenced by both preclinical and clinical investigations (Tables 1, 2). This is particularly underscored by the recent FDA approval of a novel GVHD therapy utilizing BM-MSCs. However, the exact therapeutic mechanism remains elusive, and further prospective studies are needed to confirm the effectiveness of MSCs in GVHD treatment.

**Fig. 2:**
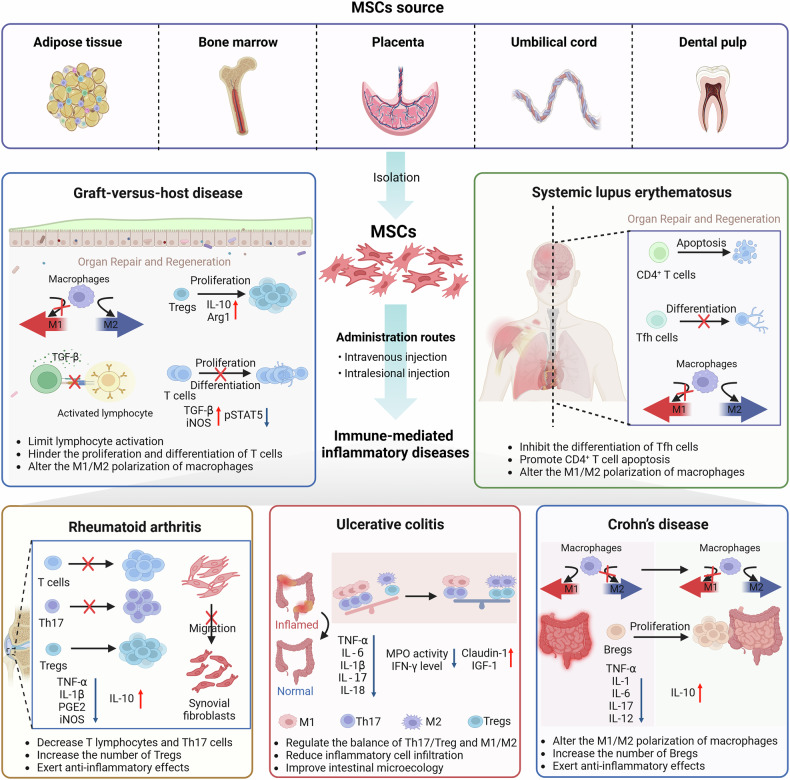
Mechanisms of MSC therapy for the treatment of immune-mediated inflammatory diseases. MSCs sourced from adipose tissue, bone marrow, placenta, umbilical cord, and dental pulp have been utilized in the treatment of graft-versus-host disease, systemic lupus erythematosus, rheumatoid arthritis, ulcerative colitis, and Crohn’s disease. The primary mechanisms of action involve the inhibition of T lymphocyte proliferation and activation, enhancement of Treg and Breg populations, reduction of proinflammatory cytokines, elevation of anti-inflammatory cytokines, and mitigation of oxidative stress and DNA damage. The figure was created with BioRender.com. M1 M1-like macrophages, M2 M2-like macrophages, Tregs regulatory T cells, Tfh cells T follicular helper cells, Th cells helper T cells, and Bregs regulatory B cells

MSCs play an important role in immune regulation (Fig. 2). Transforming growth factor beta (TGF-β) secreted by BM-MSCs plays a key role in inducing regulatory T cell (Treg) generation and limiting lymphocyte activation. Wu et al.^71^ demonstrated that TGF-β1 gene-modified BM-MSCs could transform MΦs into an anti-inflammatory M2-like phenotype. This transformation increases the proportion of Tregs and the secretion of proteins, such as IL-10 and arginase 1 (Arg1). As a result, it regulates the immune response and reduces the severity of aGVHD. According to a study conducted by Robles et al.^72^, the administration of human BM-MSCs through the tail vein of mice was found to increase the production of inducible nitric oxide synthase (iNOS), inhibit signal transducer and activator of transcription 5 (STAT5) phosphorylation, decrease T-cell proliferation, and increase the number of CD3^+^CCR7^+^ cell populations. The aryl hydrocarbon receptor is a transcription factor activated by ligands that modulates the immunomodulatory activities of BM-MSCs by ubiquitinating eukaryotic elongation factor 2 kinase, thereby regulating nitric oxide levels through iNOS synthesis.^73^ These findings suggest that this approach leads to more efficient immune suppression. In another study, Wang et al.^74^ revealed that UC-MSCs expressed high levels of C-X-C motif ligand 1 (CXCL1), leading to the accumulation of myeloid-derived suppressor cells, which attenuated the incidence and severity of GVHD. Human P-MSCs downregulated the GVHD-induced redox imbalance in erythrocytes. In addition, the mechanism by which human P-MSCs mediate the increase in the antioxidant capacity of erythrocytes involves the upregulation of glucose metabolism.^75^

BM-MSCs genetically modified with AKT1 have a survival advantage and an enhanced immunomodulatory function in liver GVHD both in vitro and in vivo.^76^ Another experiment revealed that KMRC011-primed MSCs had enhanced immunosuppressive effects on the proliferation of lymphocytes. The macrophages obtained from the spleens of mice treated with KMRC011-primed MSCs presented an enhanced anti-inflammatory M2 phenotype.^77^ CEACAM1-modified MSCs effectively inhibited the proliferation and activation of T cells and the inflammatory responses of monocytes in a GVHD mouse model.^78^ However, the response to MSC infusion may be disease specific. For example, infused IFNγ-pretreated MSCs effectively protected mice against lethal acute radiation syndrome but failed to protect mice with GVHD.^79^ It has been reported that co-transplanting MSCs with hematopoietic stem cells reduces the incidence of aGVHD; however, many results have shown no significant difference compared with the historical control group.^80^

EVs play a crucial role in cellular communication.^81^ They reprogram target cells, regulate normal physiological processes, and influence pathological conditions. Moreover, EVs act as carriers of genetic information, making them valuable agents in various biological processes and potential therapies. BM-MSCs-EVs, which are extracellular vesicles derived from BM-MSCs, have the ability to impact the activation of macrophages,^82^ hinder the proliferation and differentiation of T cells and B cells,^83,84^ and reduce the secretion of proinflammatory cytokines.^85^ Additionally, they can stimulate the production of anti-inflammatory cytokines. Systemic infusion of BM-MSCs-EVs in a mouse model of aGVHD can reduce pathological damage to multiple organs targeted by aGVHD and prolong animal survival.^86^ This protective effect is believed to be attributed to the ability of BM-MSC-EVs to inhibit the proliferation of CD4^+^ and CD8^+^ T cells, as well as the ability of naive T cells to differentiate into effector T cells, thus retaining peripheral naive T cells and CD4^+^CD25^+^Foxp3^+^ Tregs. In contrast, normal human dermal fibroblast-derived EVs did not improve aGVHD symptoms in mice. EVs derived from MSCs modified with interferon-gamma (IFN-γ), tumor necrosis factor-alpha (TNF-α), and IL-1β increased the immunosuppressive effects on activated T cells, increased the induction of Tregs, decreased the scores and prolonged the survival time in a mouse GVHD model.^87^

These findings suggest that the immunosuppressive mechanism triggered by MSCs may involve a combination of both cell‒cell contacts and soluble factors. With respect to the mechanism of treating GVHD, the primary focus should be on the immunomodulatory function of MSCs, with a subsequent emphasis on inhibiting the inflammatory response. Additionally, further studies should explore the functions of MSCs in the tissue repair of GVHD target organs, as well as their role in promoting hematopoietic reconstruction and vascular regeneration.

### Systemic lupus erythematosus

Systemic lupus erythematosus (SLE) is a long-lasting autoimmune disorder characterized by dysfunction in various organs. The abnormal activation of immune cells, such as B cells, T cells, macrophages, basophils, and DCs, significantly contributes to both the onset and progression of SLE.^88^ Currently, the conventional treatments for SLE involve the use of corticosteroids and immunosuppressants.^89^ Importantly, immunosuppressive therapy fails to adequately prevent disease recurrence in more than 50% of patients, and in certain instances, high-dose regimens may actually increase the risk of severe infections and mortality.^90,91^ In recent years, MSCs have arisen as potential innovative treatments for SLE because they have the ability to target T cells and reduce the symptoms associated with SLE (Table 2).

Numerous studies have been conducted to evaluate the mechanism and therapeutic effects of BM-MSCs in SLE (Fig. 2). Zhou et al.^92^ conducted a study in which they reported that treatment with BM-MSCs resulted in a reduction in T lymphocyte proliferation, anti-double-stranded DNA (ds-DNA) antibody levels, serum creatinine, and 24-hour protein urea levels. MSCs mediated immunomodulatory factors CXCL5 and anti-CCL24 to ameliorate GVHD by reducing the proliferation of Th 1 and Th 17 and cytotoxic T lymphocytes and natural killer cells to increase mouse immunosuppressive neutrophils.^93^ The contact kinetics observed between BM-MSCs and T cells emphasize the crucial importance of cell‒cell interactions in the inhibition of T-cell activation by BM-MSCs. This discovery offers the potential for developing innovative therapeutic strategies for individuals with SLE. Human UC-MSC transplantation ameliorated the symptoms of lupus and increased the senescence of splenic CD4^+^ T cells through silent information regulator sirtuin 1 (Sirt1)/p53 signaling via miR-199a-5p in MRL/*lpr* mice.^94^ Additionally, UC-MSCs alleviate lupus symptoms and reverse immune imbalance by promoting CD4^+^ T-cell apoptosis,^95^ reducing the number of proinflammatory central memory CD4^+^ T cells, and increasing the number of anti-inflammatory CD8^+^ T cells and anti-inflammatory resident macrophages.^96^ UC-MSCs can inhibit respiratory mitochondrial biogenesis in activated T cells to downregulate autophagy and consequently decrease T-cell apoptosis through mitochondrial transfer.^97^ BM-MSCs and UC-MSCs can inhibit the differentiation of T follicular helper cells (Tfh cells).

MSCs might ameliorate SLE by interacting with various cell types. Specifically, UC-MSCs downregulated CD11c expression in DCs and suppressed the levels of proinflammatory cytokines, such as TNF-α, IFN-γ, and IL-6, while increasing IL-10 levels.^98^ Furthermore, UC-MSC transplantation has been shown to ameliorate both atherosclerosis and SLE by reducing the number of myeloid-derived suppressor cells through the secretion of PGE2.^99^ UC-MSCs prevent podocyte injury, possibly by reducing macrophage infiltration and polarizing macrophages toward an anti-inflammatory phenotype.^100^ In murine models of lupus, UC-MSCs improved survival and renal function by targeting splenic neutrophils and macrophages, leading to reductions in IL-6 and CXCL1 and stabilizing the levels of interferon-γ and TNF-α.^101^ Moreover, UC-MSCs alleviate SLE by promoting CD206 expression and enhancing the phagocytic activity of macrophages in an IL-6-dependent manner.^102^ DPSCs and periodontal ligament stem cells (PDLSCs) have also demonstrated efficacy in alleviating symptoms in SLE mouse models, with DPSCs being notably effective in reducing kidney glomerular lesions and perivascular inflammatory infiltration.^103^

In recent years, research focused on treating SLE with BM-MSCs has shifted toward B-cell inhibition and dual inhibition of T and B cells. Studies have shown that MSCs can effectively inhibit B-cell proliferation, differentiation, and chemotaxis.^104–107^ UC-MSC transplantation inhibited the proliferation and differentiation of peripheral blood B cells in the early stages in MRL/lpr mice.^108^ A reduction in CCL2 expression in the BM-MSCs of SLE patients impaired their ability to inhibit B cells. However, the overexpression of CCL2 can restore the immunosuppressive ability of B cells.^109^ Human gingiva-derived mesenchymal stem cells (G-MSCs) limit the development of autoantibodies and proteinuria and decrease lupus nephritis scores by suppressing B-cell activation, proliferation, and differentiation.^110^

The infusion of BM-MSCs could directly prevent naive CD4^+^ T cells from differentiating into Tfh cells. Furthermore, it decreases the numbers of Tfh cells (CD4^+^CXCR5^+^PD-1^+^) and their circulating precursors (CD4^+^CD44^+^CXCR5^+^PD-1^+^) and reduces the presence of germinal center (GC) B cells (B220^+^GL7^+^) and plasmacytes (B220^lo^ CD138^+^). In addition, it minimizes the infiltration of anti-dsDNA antibodies in the serum of mice, as well as the presence of CD4^+^ T cells and B220^+^ B cells in the kidneys.^111^ These results indicate that chemokines are essential for modulating the immune response of BM-MSCs toward B cells and T cells. Treatment with chemical substances, such as phorbol myristate (PMA), can enhance the ability of human BM-MSCs to inhibit B cells. Lee et al.^112^ conducted a study in which they treated human BM-MSCs with phorbol myristate acetate, which reduced the influx of T cells, B cells, macrophages, and DCs to the kidneys. These findings suggest that chemical methods could enhance the activity of human BM-MSCs for the treatment of SLE patients.

### Rheumatoid arthritis

Rheumatoid arthritis (RA) is a chronic autoimmune disease characterized by chronic inflammatory synovitis and the destruction of articular cartilage and bone tissue. This destructive process can result in irreversible joint damage and loss of mobility, significantly impacting patients’ quality of life and overall lifespan.^113^ At present, glucocorticoids, immunosuppressants, and biological agents are the primary treatment methods for RA. However, these methods have limited efficacy, and prolonged usage can lead to severe adverse reactions.^114^ The use of MSCs has emerged as a promising avenue of research in the field of RA treatment owing to their anti-inflammatory, immunomodulatory, and paracrine effects and ability to differentiate into multiple cell types (Table 2 and Fig. 2).

In rats with collagen-induced arthritis (CIA), BM-MSC transplantation has been shown to inhibit the expression of cytokines associated with bone destruction, alleviate joint inflammation, and inhibit synovial proliferation and cartilage destruction.^115^ UC-MSCs also downregulate proinflammatory cytokines and osteoclastogenesis while enhancing the levels of inflammatory mediators in the ankle joints of mice.^116^ Intra-articular injection of BM-MSCs overexpressing C-X-C chemokine receptor type 7 (CXCR7) in CIA rats results in the production of anti-inflammatory factors via regulation of the WNT, Hedgehog, and Notch signaling pathways.^117^ This enhances bone and cartilage differentiation, thus improving ankle joint swelling, joint deformity, joint destruction, and cartilage damage in rats. Moreover, transplanting BM-MSCs with IL-4 effectively reduced synovitis and systemic inflammation in a CIA model, as evidenced by substantial reductions in CRP, antinuclear antibodies, TNF-α, and monocyte chemoattractant protein-1.^118^ TNF-α, IL-10, iNOS, matrix metalloproteinase 9 (MMP-9), and TGF-β1 are important targets that mediate the anti-arthritic effect of BM-MSCs.^119^ BM-MSCs effectively reduce TNF-α, CD83, CCR7, and MIP-1β protein levels in myeloid DCs and all monocyte subsets.^120^ Both BM-MSCs and indomethacin (IMC) reduce the levels of proinflammatory cytokines and increase the levels of the anti-inflammatory cytokine IL-10 in mice suffering from arthritis induced by complete Freund’s adjuvant (CFA). This treatment also reduces the concentration of anti-cyclic citrullinated peptide (CCP), decreases synovial degeneration, and inhibits paw swelling.^119^

Injection of BM-MSC-derived exosomes (BM-MSC-Exos) into RA mice was found to reduce the secretion of inflammatory cytokines and alleviate RA progression.^121^ BM-MSC-Exos carrying miR-150-5p have been shown to downregulate the expression of MMP14 and vascular endothelial growth factor (VEGF), leading to the inhibition of synovial fibroblast migration, invasion, and angiogenesis, ultimately reducing ankle joint damage.^122^ Furthermore, BM-MSC-EVs carrying miR-34a have been shown to regulate fibroblast function and alleviate RA inflammation by inhibiting cyclin I expression and activating the ATM/ATR/p53 signaling pathway.^123^ Notably, BM-MSC-EVs can effectively transfer cell-targeting drugs and biomolecules.^124^ For example, BM-MSC-EVs can transfer miR-21 and target ten-eleven translocation 1 (TET1) to inhibit Kruppel-like factor 4 (KLF4) expression, promote fibroblast-like FLS proliferation, and downregulate the expression of inflammatory factors, such as IL-1β, TNF-α, PGE2, NO, and iNOS.^125^ This reduces inflammatory infiltration and synovial hyperplasia and increases bone mineral density (BMD). Recent studies have also examined the use of other MSC-EVs for RA treatment. Tsujimaru et al.^126^ revealed that AD-MSC-EVs improved RA in the IL-1Ra knockout mouse model. Furthermore, joint swelling was reduced by AD-MSCs and AD-MSC-EVs, and these effects were associated with decreased proinflammatory cytokines, which might be mediated by the suppression of helper T (Th) cells. Human UC-MSC-EVs ameliorate CIA via the inhibition of synovial hyperplasia via the inhibition of T lymphocyte proliferation and the promotion of T lymphocyte apoptosis while decreasing the proportion of Th17 cells and increasing that of Tregs.^127^

Currently, the majority of research on the treatment of RA has focused primarily on reducing synovitis, arthritis, cartilage, and joint destruction. However, research on cartilage repair, bone tissue repair, microangiogenesis, and pannus formation is limited. It is crucial to strengthen research efforts in these areas to explore the therapeutic potential of MSCs. Additionally, combining MSCs with other factors or drugs may increase their efficacy against RA. Safety concerns associated with the sources of MSCs, such as UC-MSCs, also need to be addressed.^128^ Further studies are needed to determine the optimal dosage, administration method, and detailed protective mechanism of combination therapy.

### Ulcerative colitis

Inflammatory bowel disease (IBD) is a chronic, idiopathic disease that affects the gastrointestinal tract. It is characterized by symptoms such as abdominal pain, diarrhea, and rectal bleeding.^129^ The two main types of IBD are ulcerative colitis (UC) and Crohn’s disease (CD). The pathogenesis of IBD remains unclear, and there are currently no specific treatments available. Therefore, it is crucial to find improved strategies for both preventing and treating IBD. MSCs have become a new and promising method for the treatment of IBD because of their unique abilities of homing, immune regulation, self-renewal, and tissue repair (Tables 1 and 2).

UC is a chronic, nonspecific inflammatory condition that begins in the rectal mucosa and extends continuously to the colon mucosa. The main symptom of UC is bloody diarrhea, and the disease follows a clinical course marked by alternating periods of worsening symptoms and periods of improvement. The main goals of medical management involve quickly attaining a clinical response, normalizing biomarkers, and then sustaining clinical remission while achieving endoscopic normalization to avert long-term disability.^130^ Despite the availability of various treatments, 10–20% of patients continue to require proctocolectomy due to refractory disease. In pursuit of more effective treatment alternatives, researchers have progressively focused their efforts on the use of MSCs as a promising therapeutic strategy.

In inflammatory diseases, increased inflammation level can have varying effects on stem cell differentiation, regeneration, and wound healing processes.^131^ The intraperitoneal injection of 1 × 10^6^ human BM-MSCs into IBD mice resulted in thrombospondin-1 (THBS1)-dependent TGF-β activation, the induction of peritoneal IL-10^+^ Bregs, the downregulation of MPO activity, the reduction in IFN-γ and TNF-α levels, the minimal infiltration of inflammatory cells within the lamina propria, and improvements in weight loss and colon shortening in mice.^132^ Intravenous infusion of human embryonic MSCs alleviated colitis in mice by increasing the circulating insulin-like growth factor 1 (IGF-1) level. Increased IGF-1 maintains the integrity of epithelial cells and contributes to their repair and regeneration.^133^

MSCs mitigate colitis by exerting potent immunomodulatory effects. MSCs derived from human gingiva also have immunomodulatory functions and ameliorate inflammation-related tissue destruction in mice with colitis.^134^ AD-MSCs may alleviate ulcerative colitis by communicating with macrophages to block inflammation.^135^ BM-MSCs produce immunoregulatory molecules, including TSG6, that reduce intestinal inflammation in mice with colitis.^136^ Moreover, they promote the proliferation of intestinal epithelial cells and the differentiation of intestinal stem cells by downregulating both Th1-Th17-driven autoimmune and inflammatory responses and by increasing Th2 activity.^137^ AD-MSCs have also been demonstrated to have immunosuppressive effects, inhibiting the proliferation of peripheral blood mononuclear cells (PBMCs) and the production of IFN,^138^ reducing the infiltration of M1 macrophages.^139^

Preclinical and clinical studies have highlighted the sustained efficacy of MSC therapy in promoting long-term effects in patients with Crohn’s disease. In DSS-induced murine colitis, a single dose of AD-MSCs confers long-term protection by reprogramming innate immunity, increasing the number of M2 macrophages that mitigate recurrent inflammation, even in adaptive immune-deficient mice.^140^ Clinically, allogeneic BM-MSCs achieved 70% fistula closure in patients with Crohn’s disease at 4 years,^141^ whereas autologous AD-MSCs maintained 75% closure at 2 years.^142^ These findings underscore MSC-driven immune modulation as a durable strategy for inflammation resolution, particularly in refractory IBD, where conventional therapies fail to address persistent immune dysregulation.

The pathogenesis of UC is closely associated with the intestinal microbiota and the intestinal barrier.^143^ Exos and Evs play crucial roles in immune cell recruitment. In a study carried out by Gu et al.^144^ the effects of BM-MSC-Exos miRNA-181a on the intestinal microbiota, immune response, and intestinal barrier function of UC patients were investigated. The results showed that miR-181a could lower the concentrations of TNF-α, IL-6, IL-1β, IL-17, and IL-18, thereby reducing inflammatory cell infiltration. Additionally, it upregulates the expression of the intestinal barrier marker proteins claudin-1 and zonula occludens-1 (ZO-1), thereby improving the intestinal microecology, enhancing intestinal barrier function, and maintaining the structural integrity of the colon. Coadministration of AD-MSC-Exos with anti-IL-12 p40 significantly decreased DAI scores in a mouse model.^145^ Hypoxia-preconditioned hair follicle MSC-Exos (Hy-Exos) play a vital role in suppressing inflammatory progression in UC and suggest that miR-214-3p is effective in alleviating UC. Hy-Exos may exert these effects via miR-214-3p-mediated inhibition of the PI3K/AKT/mTOR signaling pathway and mitochondrial dynamic stability.^146^

EVs secreted by BM-MSCs are vital for their immunomodulatory effects. A study involving UC model mice showed that administering EVs via intraperitoneal injection led to a significant decrease in the phosphorylation levels of Janus kinase 1 (JAK1) and signal transducer/activator of transcription 1 (STAT1).^147^ Furthermore, Evs enhanced the activity of STAT6, promoting M2 macrophage polarization through JAK1/STAT1/STAT6 signaling. Upon the transfection of BM-MSC-EVs with Ephrin type-B receptor 2 (EphB2), the secretion of IL-6 and transforming TGF-β1 can be modulated, leading to the inhibition of STAT3 activation, a reduction in Th17 cell numbers, and an increase in Treg expression.^148^ This regulation is achieved by interfering with the JAK/STAT pathway, which ultimately leads to a balance between Th17 cells and Tregs and an improvement in colon structural disorders. The results of these two similar studies suggest that BM-MSCs improve symptoms of UC and colon pathology by influencing the JAK/STAT inflammatory signaling pathway. This effect is achieved by modulating the secretion of inflammatory factors as well as the immune functions of macrophages and T cells. MSC-EVs-PD-L1 exhibit an impressive ability to regulate various activated immune cells in an immunosuppressed state.^149^ PD-L1-EVs mitigate colonial inflammation, apoptosis and oxidative stress by blocking the activation of the PI3K/Akt/mTOR pathway and regulating the balance of Th17/Treg cells.^150^

Thus, MSCs play crucial roles in mitigating oxidative stress, increasing the production of anti-inflammatory cytokines, concurrently reducing proinflammatory cytokine levels, and modulating the balance of macrophages and Th17/Treg cells. These mechanisms effectively alleviate symptoms of UC and reduce pathological damage to the intestinal mucosa (Fig. 2).

### Crohn’s disease

CD represents long-lasting inflammation of the gastrointestinal tract that is often linked to severe complications, such as fistula formation and bowel obstruction. Treating CD can be challenging due to the presence of complications, such as fibrotic stenosis, perianal fistula, intestinal obstruction, perforation, and toxic colon dilation.^151^ Although long-term oral medications are frequently prescribed, they may cause side effects, including headache, nausea, vomiting, and possible toxicity to the liver and kidneys.^151^ Surgical interventions also carry risks, including anal incontinence and healing complications, as well as the possibility of adverse events and disease recurrence. Therefore, exploring safer and more effective treatment approaches that can minimize the recurrence rate is imperative.

Compared with subcutaneous tissue-derived cell or EV therapy, the capacity of MSCs and their derived EVs isolated from Crohn’s disease patients to alleviate DSS-induced colitis is lower.^152^ A study revealed that mixed cytokines mimicking the local condition of inflamed UC colonic or CD fistulas differentially affected the immunomodulatory and tissue regenerative characteristics of MSCs.^153^ BM-MSCs exposed to patient PBMCs undergo apoptosis and release PGE2.^154^ However, Anbazhagan et al.^155^ revealed that MSCs from IBD patients had normal transcriptional and immunomodulatory properties with therapeutic effects.

The effects of BM-MSCs on IBD have been demonstrated to be mediated by various immune cells, particularly Bregs (Fig. 2). However, the specific characteristics and functions of Breg subsets are still not well understood. A novel subset of Bregs, identified by the presence of CD23 and CD43, was confirmed by Chen et al.^156^ are involved in the immunomodulatory functions of human BM-MSCs. In a trinitrobenzene sulfonic acid-induced colitis mouse model, the intraperitoneal administration of 1×10^6^ human BM-MSCs led to an increase in the number of CD23^+^ CD43^+^ Bregs and IL-10. The injection of human BM-MSCs also inhibited the secretion and proliferation of inflammatory cytokines in T cells, leading to decreases in the levels of TNF-α, IFN-γ, IL-1β, IL-6, and IL-17. Furthermore, it improved colon mucosal destruction and submucosal edema and restored colon length. These findings align with the findings of Xie et al.,^157^ who reported a decrease in TNF-α, IL-12, and VEGF levels; an increase in IL-10 levels; and increases in transmural inflammation, epithelial cell exhaustion, disseminated fibrosis, local loss of crypts, colon thickness, and weight gain in CD mice following AD-MSC or BM-MSC treatment.

Human BM-MSCs significantly reduce oxidative stress and inflammation in IL-10 KO mice.^158^ Human BM-MSCs mediate these effects via the modulation of T cells and macrophages, and the long-term efficacy of BM-MSCs in mediating anti-inflammatory macrophages occurs via efferocytosis.^159^ P-MSCs, which are associated mainly with tyrosine metabolism, can also be used to treat CD mice effectively on the basis of serum metabolomics results.^160^ UC-MSCs can improve CD scores and attenuate the polarization of M1 macrophages.^161^

AD-MSCs treated with IFN-γ and kynurenic acid (KYNA) significantly upregulated the expression and secretion of IDO-1, which effectively ameliorated colitis injury and fibrosis.^162^ When engineered to overexpress HIF-1α and telomerase (MSCs-T-HIF) and conditioned with proinflammatory stimuli, EVs (EV_MSC-T-_^HIFCs^) with potent immunomodulatory activity are released. It promoted healing in a mouse colitis model by preserving colon length and intestinal mucosa architecture and altering the ratio of M1/M2 infiltration.^163^

BM-MSC-EVs transfected with miR-378a-3p inhibited the peroxisome proliferator-activated receptor α (PPAR α) signaling pathway by decreasing the expression of GATA-binding protein 2 (GATA2) and downregulating aquaporin 4 (AQP4). This leads to an increase in body weight and colon length in mice, as well as a reduction in inflammatory cell infiltration, crypt loss, mucosal destruction, and edema in the colon mucosa.^164^ Recently, Li et al.^165^ designed an EV-biodegradable nanofiber-hydrogel composite (NHC), which effectively reduced inflammation at the fistula site and promoted tissue healing and regeneration via macrophage polarization and neovascularization. The enhanced therapeutic results contributed to the extended retention and sustained release of EVs accomplished by NHCs. Additionally, UC-MSC-Exos could attenuate mesenteric inflammation and colitis by promoting mesenteric macrophage M2 polarization.^166^

In summary, MSCs derived from adipose tissue and bone marrow have similar phenotypic characteristics, but the procurement of AD-MSCs is easier. AD-MSCs may be a better source of MSCs for cell therapy for CD.^157^ Notably, most studies have focused on the multidirectional differentiation, immune regulation, and tissue repair functions of MSCs. More studies should be carried out to explore their ability to promote intestinal epithelial cell regeneration and tissue repair. Additionally, gene-edited MSCs have gained significant attention and hold promise as a new treatment method for CD.

## MSCs and respiratory diseases

### Acute lung injury

The lung is perpetually subjected to pathogens and potentially detrimental agents via the respiratory tract.^167^ While the lung possesses regenerative capabilities, this intrinsic repair process frequently falls short of facilitating adequate cellular renewal under pathological circumstances. Acute lung injury (ALI), acute respiratory distress syndrome (ARDS), and novel coronavirus pneumonia (COVID-19) can cause significant damage to the lungs, which is challenging to repair.^168,169^ Cell therapy is an emerging field in the treatment of lung diseases, where BM-MSCs provide a promising avenue for treatment. Research has demonstrated that MSCs and their derivatives play crucial roles in regulating tissue damage by controlling the inflammatory response, promoting tissue repair and regeneration, and exerting a reparative effect on lung damage.

ALI refers to a state marked by acute inflammation and the possible development of fibrosis in lung tissue.^170^ It involves an excessive inflammatory response in the lungs and breakdown of the alveolar‒capillary barrier, resulting in the accumulation of high-protein edema fluid and the production and secretion of proteases and toxic mediators.^171^ Despite the progress made in therapeutic interventions that have improved survival rates for individuals with ALI, the prognosis remains bleak.^172^ In recent years, evidence has suggested that MSCs can improve ALI and pulmonary fibrosis. Researchers have focused on the efficacy and mechanism of MSC application in animal ALI models.

MSCs have been shown to reduce the inflammatory response through various mechanisms, which causes a decrease in proinflammatory cytokines and pathological lung damage in ALI models. Ortiz et al.^173^ reported that systemically administered mouse BM-MSCs could engraft into recipients with bleomycin-induced lung injury. Moreover, the results revealed that these MSCs were located in the lung area and exhibited an epithelium-like morphology, reducing inflammation and collagen deposition in the lung. In addition, BM-MSCs protected lung tissue from ALI by blocking TNF-α and IL-1. IL-1 receptor antagonist-expressing BM-MSCs may provide a novel tool for treating ALI.^174^ UC-MSCs ameliorate burn-induced ALI by promoting TSG-6 secretion and inhibiting inflammatory reactions in lung tissue.^175^ Allogeneic administration of UC-MSCs decreased the lung injury score, protected the alveolar structure, inhibited neutrophil infiltration into the lung interstitium, and stimulated cytoprotective cytokine production in the lung.^176^ UC-MSCs attenuate ALI by redefining the gut and lung microbiota.^177^ Fecal microbiota transplantation (FMT) from human UC-MSC-treated mice alleviated ALI by inhibiting inflammation and reconstructing the gut microbiota; additionally, the TLR4/NF-κB and Nrf2/HO-1 pathways were involved in the improvement of ALI by FMT.^178^

Wang et al.^179^ designed a microscopic imaging technique, which provides a method for real-time monitoring of MSCs. These findings revealed that increased VCAM1 in endothelial cells could enhance MSC retention in the lung, further ameliorating acute lung injury. BM-MSC treatment could affect the subtype distribution and function of neutrophils. BM-MSCs upregulate the expression of cluster of differentiation 24 (CD24), shifting activated neutrophils to aged neutrophils, thereby inhibiting inflammation by reducing reactive oxygen species (ROS) production and NADPH oxidase.^180^ BM-MSCs have the potential to increase the bioenergetics of damaged alveolar cells by providing them with fresh mitochondria.^181^ This, in turn, promoted self-repair, healing, and regeneration of damaged lung tissue, leading to improved ALI. Moreover, BM-MSCs inhibited the inflammatory and phenotypic transformation of alveolar macrophages, thereby affecting the inflammatory phenotype of lung organoids through the NF-κB pathway.^182^ In the paraquat-induced ALI model, combined mitigation of the NF-κB and IL-17 signaling pathways in BM-MSC-transplanted samples was observed.^183^ The downregulation of MyD88-NF-κB signaling protein expression was also demonstrated in human UC-MSC treatment.^184^ Furthermore, research has indicated that BM-MSCs reduce the influx of CD8^+^ T cells within the intestinal region. Additionally, they lowered the levels of proinflammatory cytokines such as IFN-γ, TNF-α, IL-1α, IL-1β, and IL-6 by promoting the expression of the tight junction protein ZO-1 on the ileal membrane. This ultimately led to a reduction in lung and intestinal damage in ALI mice.^185^ Lung-resident MSCs attenuate lipopolysaccharide (LPS)-induced ALI by increasing the balance of Tregs and Th17 cells.^186^

When administered after ALI induction, MSCs-EVs exhibit high lung accumulation and are associated with reduced inflammation, as evidenced by decreased neutrophil influx and macrophage inflammatory protein-2 levels.^187^ Human UC-MSCs primed with IFN-γ-Exos reduce oxidative stress and inflammatory responses in mice,^188^ and MSC-Exos reverse ALI through the nuclear factor NF-E2-related factor 2 (Nrf-2)/antioxidant response element (ARE) and NF-κB signaling pathways.^189^ The therapeutic effects of MSC-EVs are compromised when keratinocyte growth factor (KGF) is knocked down, and Nrf2 knockdown diminishes their anti-inflammatory and antioxidant activities in LPS-stimulated macrophages.^187,190^ Intratracheal administration of MSC-EVs reduces virus shedding in nasal swabs, influenza virus replication in the lungs, and virus-induced production of proinflammatory cytokines in influenza-infected pigs.^191^ Treatment with UC-MSC-EVs improves survival rates in ALI mice by alleviating pulmonary microvascular permeability and inhibiting proinflammatory responses, including reductions in TNF-α, IL-1β, IL-6, and macrophage chemoattractant protein-1 (MCP-1). Notably, miR-223-3p acts as a critical mediator of UC-MSC-EV-induced regulatory effects by inhibiting poly(adenosine diphosphate-ribose) polymerase-1 (PARP-1) in lung epithelial cells.^192^ EVs and Exos can also interact with lncRNAs. MSC-EVs enhance autophagy and ameliorate ALI, in part through the delivery of miR-100.^193^ MSC-Exos inhibited the EMT process in LPS-induced MLE-12 cells by transmitting miR-182-5p and miR-23a-3p to target Ikbkb and Usp5, respectively.^194^

MSC infusion can protects alveolar epithelial cells and promotes neovascularization, preventing lung fibrosis. BM-MSCs alleviate ALI via paracrine hepatocyte growth factor (HGF) by inducing mature DCs to differentiate into regulatory DCs.^195^ Human dermal MSCs (D-MSCs) primed with O-cyclic phytosphingosine-1-phosphate (cP1P) decreased the infiltration of macrophages and neutrophils and the release of TNF-α, IL-1β, and IL-6. In addition, p-STAT1 and iNOS levels are decreased in the lungs of human D-MSC-cP1P-treated mice.^196^ Besides, MSCs primed with BMP2 increase the level of IDO1, and the number of Foxp3^+^CD25^+^ Tregs among CD4^+^ cells is further increased in ALI mice.^197^ PGE2-primed P-MSCs reduce the severity of ALI in mice by modulating macrophage polarization and cytokine production.^198^

**Fig. 3:**
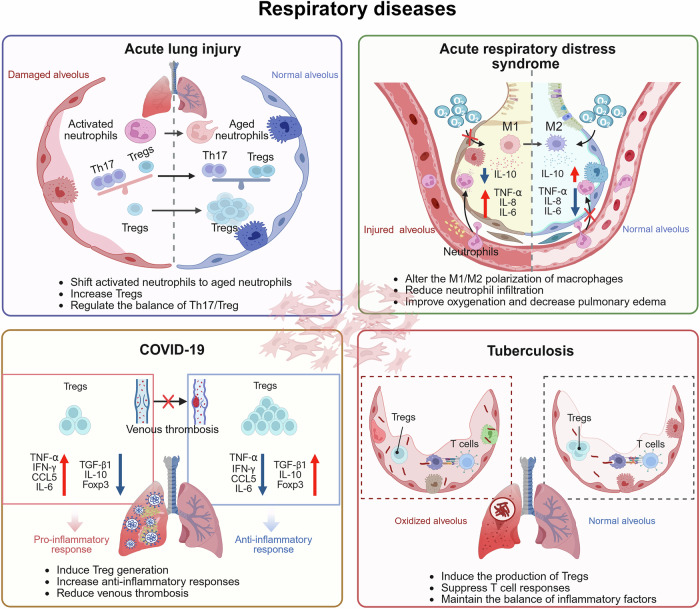
Mechanisms of MSC therapy for the treatment of respiratory diseases. MSCs sourced from adipose tissue, bone marrow, placenta, the umbilical cord, and the dermis have the potential to be administered for the treatment of acute lung injury, acute respiratory distress syndrome, COVID-19, and tuberculosis. The primary mechanisms of action involve modulating T-cell activities, attenuating proinflammatory cytokine levels, increasing anti-inflammatory cytokine production, and diminishing neutrophil infiltration. The figure was created with BioRender.com. Foxp3 Forkhead box P3, IL-6 interleukin-6, IL-8 interleukin-8, IL-10 interleukin-10, TNF-α tumor necrosis factor-alpha, TGF-β1 transforming growth factor-beta 1, CCL5 C-C chemokine ligand 5, IFN-γ interferon-gamma, M1 M1-like macrophages, M2 M2-like macrophages, Tregs regulatory T cells

These studies have confirmed the effectiveness of MSCs and their derivatives in experimental animal studies of ALI. This effectiveness can be attributed to their repair properties, which include immune regulation, alveolar epithelial and endothelial repair, and inhibition of fibrosis (Fig. 3). MSCs can exhibit either beneficial or detrimental effects depending on the microenvironment during administration.^199^ While MSCs have been shown to mitigate ventilator-induced lung injury, they can also aggravate lung fibrosis in the context of acid-primed injury. Notably, the pre-administration of glutathione peroxidase-1 prior to MSC infusion has been demonstrated to counteract these negative outcomes. In addition, which form of MSC might be the best treatment remains controversial. Ren et al.^200^ found that UC-MSCs exhibited greater protection in lung tissue repair than menstrual blood-derived MSCs did. Furthermore, research involving the use of MSCs in murine and cynomolgus monkey models has been conducted. These findings indicate that the intravenous administration of MSCs at repeated doses is safe and results in no significant systemic toxicity.^201,202^ Significant advancements have been made in various preclinical animal models, and numerous clinical trials utilizing different MSCs for the treatment of ALI have been initiated globally in recent years. The clinical application of MSCs in the management of ALI is expected to gain momentum.

### Acute respiratory distress syndrome

Acute respiratory distress syndrome (ARDS) is a prevalent critical pulmonary disease with the potential to cause respiratory failure.^203^ Its pathogenesis is associated with unregulated inflammatory cascades and increased pulmonary endothelial and epithelial cell permeability. The treatment of ARDS is still limited to supportive treatment, and there is a lack of effective drug treatment options (Fig. 3). Recently, MSCs have emerged as promising candidates for treating ARDS, and investigating their therapeutic mechanisms could offer novel insights into the management of this condition. BM-MSCs have been demonstrated to mitigate inflammation, rebuild the capillary‒alveolar membrane, and contribute significantly to the repair process of lung injury caused by ARDS.^204,205^ Autologous transplantation of BM-MSCs into the lungs of New Zealand rabbits with ARDS has been shown to decrease the levels of TNF-α and IL-6 while increasing the level of IL-10.^206^ This transplantation technique also has the ability to ameliorate symptoms associated with inflammatory cell infiltration, pulmonary hemorrhage, and pulmonary edema. These results underscore the crucial importance of transplanting BM-MSCs in restoring lung injury caused by ARDS.

The cholinergic anti-inflammatory pathway (CAP) is responsible for regulating inflammatory responses and has the potential to be used as a therapeutic target for inflammatory diseases. Acetylcholine can modulate CAP signaling within macrophages, particularly through the JAK2/STAT3/NF-κB pathway, which consequently reduces the synthesis of proinflammatory cytokines. In an ARDS mouse model, the MSC-mediated mechanism of CAP activation could increase the expression levels of choline acetyltransferase (ChAT) and vesicular acetylcholine transporter (VAChT) to alleviate ARDS-like syndrome.^207^ These results indicate that focusing on the CAP pathway is a potentially effective therapeutic strategy for treating ARDS and various other inflammatory lung diseases.

The mechanism by which MSCs treat ARDS is multifaceted. Human BM-MSCs improved oxygenation and decreased pulmonary edema in a sheep model of severe ARDS.^208^ Hypo- or hyperinflammatory ARDS environments may differentially influence the cytoprotective and immunomodulatory functions of MSCs. Moreover, both MSC-Hypo and MSC-Hyper reduced IL-6 and TNF-α levels in bronchoalveolar lavage fluid (BALF), and only MSC-Hyper reduced overall clinical outcomes, including weight loss and clinical scores.^209^ MSCs have the potential to mitigate ARDS and diminish pulmonary inflammation; however, the ablation of vimentin has been shown to impair the therapeutic efficacy of MSCs in ARDS. Further studies revealed that vimentin regulated MSC migration mainly through Rab7a.^210^ In addition, MSCs promote the phagocytic activity of macrophages through mitochondrial transfer.^211^ Morrison et al.^212^ found that when human BM-MSCs and monocyte-derived macrophages were cocultured followed by stimulation with LPS or bronchoalveolar lavage fluid from patients with ARDS, the expression of the anti-inflammatory macrophage marker CD206 increased, and the phagocytic ability improved. MSCs promoted an anti-inflammatory and highly phagocytic macrophage phenotype through EV-mediated mitochondrial transfer. P-MSC-EV treatment also reduces lung injury and neutrophil infiltration and improves alveolar barrier integrity.^213^ BM-MSC-EVs can effectively modify macrophage responses, even with inflammatory triggers such as hypo- and hyperinflammatory ARDS plasma.^214^ This reprogramming was achieved through the transfer of miR-181a in BM-MSC-EVs, which led to the downregulation of phosphatase and tensin homolog (PTEN) and the increase in pSTAT5 and suppressor of cytokine signaling 1 (SOCS1). As a result, macrophages are transformed into an anti-inflammatory state, leading to a reduction in the levels of TNF-α and IL-8 and an improvement in symptoms of lung injury. These results illuminate the role of the miR-181a-PTEN-pSTAT5-SOCS1 pathway in the therapeutic application of BM-MSC-EVs for treating ARDS. The exact way in which BM-MSCs and their derivatives provide therapeutic benefits in ARDS remains largely unclear. Nonetheless, it is thought to be associated with a mixture of regenerative and anti-inflammatory characteristics. BM-MSCs can mitigate lung histopathological alterations and inflammatory reactions within the ARDS inflammatory microenvironment by modulating the equilibrium between proinflammatory and anti-inflammatory macrophages.

### COVID-19

COVID-19 is an illness that can harm various organs throughout the body. This condition is induced by severe acute respiratory syndrome coronavirus 2 (SARS-CoV-2), which primarily affects the lungs. The fundamental pathophysiological process involves the attachment of SARS-CoV-2 to angiotensin-converting enzyme 2 (ACE2), which is located on the cell membrane, resulting in both localized and widespread inflammatory responses, oxidative stress, and cellular and tissue hypoxia, among other impacts.^215^ With the help of additional treatment, the majority of patients can rely on their own immune system to eliminate the virus, repair inflammatory damage, and recover. However, a small portion of patients experience severe illness and even death. Research has demonstrated that BM-MSCs have the ability to repair the inflammatory damage caused by COVID-19 because of their multipotent characteristics and immunomodulatory effects^216^ (Fig. 3). Hence, actively exploring the potential of MSC treatment for the benefit of society as a whole is highly valuable. The infusion of UC-MSCs has the potential to lower proinflammatory cytokine levels and neutrophil extracellular traps (NETs) while supporting the preservation of SARS-CoV-2-specific antibodies. Furthermore, MSC treatment regulated B-cell subsets and increased the expression of CD28 in T cells. In addition, studies in a murine model confirmed that AD-MSCs suppressed NET release and reduced venous thrombosis via upregulation of the kindlin-3 signaling pathway.^67^ Zhang et al.^217^ generated ACE2 extracellular domain-Fc and single-chain variable fragment against IL-6 receptor (scFv-IL6R)-Fc fusion proteins to neutralize viruses and ameliorate the IL-6 cytokine storm differentially.

The immune response to COVID-19 infection involves both innate and adaptive immunity. The most prevalent and severe complication associated with COVID-19 is ARDS. Kaspi et al.^218^ utilized differentiation medium to induce the directional differentiation of BM-MSCs into BM-MSC-neurotrophic factor (NTF) cells, also known as NurOwn cells, which secrete NTF. Researchers then utilized the Exos secreted by these BM-MSC-NTF cells (referred to as BM-MSC-NTF Exos) to treat ARDS. The findings of the present study revealed that treatment with either BM-MSC-Exos or BM-MSC-NTF Exos increased blood oxygen saturation, reduced neutrophil accumulation, and decreased alveolar space thickness in mice. Notably, the efficacy of BM-MSC-NTF Exos was superior to that of BM-MSC-Exos, which aligns with the superior capability of BM-MSC-NTF Exos. Research has shown that the delivery of BM-MSC-NTF Exos is vital for restoring the balance of the host immune response and decreasing the levels of IFN-γ, IL-6, C-C motif chemokine ligand 5 (CCL5), and TNF-α. This evidence implies that BM-MSC-NTF Exos could have considerable promise in addressing lung injury and enhancing the outcomes of patients with COVID-19. The expression levels of TGF-β1, IL-10, and forkhead box protein 3 (FOXP3) were significantly increased in AD-MSC-Exo-treated PBMCs. AD-MSC-Exos have the potential to increase anti-inflammatory responses via the induction of Tregs.^219^

Studies investigating the treatment of MSCs with SARS-CoV-2 suggest that their therapeutic function largely stems from their ability to modulate the immune response, thereby mitigating overactivation of the immune system in infected individuals. Nevertheless, additional clinical research is essential to gain deeper insight into their therapeutic effects and safety.

### Tuberculosis

Tuberculosis (TB) represents a significant global public health challenge and is caused primarily by *Mycobacterium tuberculosis* (MTB).^220^ The treatment options for individuals diagnosed with TB frequently involve drug-resistant or multidrug-resistant strains, and the pharmacological agents currently utilized in clinical settings are limited in their efficacy against MTB, often resulting in numerous adverse effects. Furthermore, anti-TB medications can cause considerable hepatotoxicity, leading to drug-induced liver injury and, in some cases, necessitating permanent cessation of treatment.^221^ Consequently, TB is a condition that demands innovative therapeutic approaches.

MSCs are observed in and around human tuberculosis granulomas harboring MTB bacilli.^222^ MTB can be successfully hidden in BM-MSCs partly through the nucleotide oligomerization domain-2 (NOD-2) and Toll-like receptor (TLR-4) signaling pathways, and targeting MTB through NOD-2 and TLR-4 can be a useful method to eliminate the MTB concealed inside MSCs.^223^ Das et al.^224^ reported that MTB maintained long-term intracellular viability within human CD271^+^/CD45^−^ BMSCs, which might be a therapeutic target for MTB persistence. Muraviov et al.^225^ established the experimental rabbit kidney TB model by injecting MTB H37Rv (10^6^ CFU); after the BM-MSCs were transplanted, the level of inflammatory proteins decreased, the area of specific and destructive inflammation in the kidneys decreased, and the formation of mature connective tissue increased. During MTB infection, MSCs induce the production of FoxP3^+^ Tregs, which may help establish T-cell tolerance. NO produced by MSCs might also play a critical role in suppressing T-cell responses.^226^ In general, MSCs play a significant role in maintaining delicate balance within granulomas containing pathogenic microorganisms without eliminating them (Fig. 3). This mechanism avoids extensive immunosuppression and instead confines immune suppression to the infected tissue.^226^

BM-MSC-EVs significantly increased the levels of anti-inflammatory cytokines and decreased the levels of proinflammatory cytokines. MSC-EVs carrying miR-20b inhibited nuclear factor of activated T cells 5 (NFAT5) and inactivated the TLR signaling pathway, thus significantly mediating the immune response.^227^ The current treatments for MTB, especially multidrug-resistant (MDR-TB) and extensively drug-resistant (XDR)-TB, are costly and ineffective. Therefore, it is essential to study effective therapies. The transplantation of MSCs may be a competent therapy. More data obtained from experiments and clinical trials are key to managing indomitable MDR/XDR-TB strains.

## MSCs and skeletal diseases

**Fig. 4:**
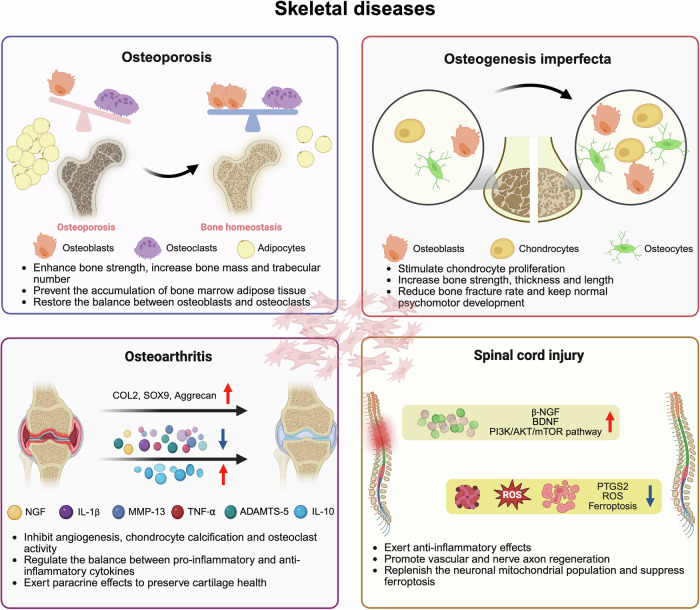
Mechanisms of MSC therapy for the treatment of skeletal diseases. MSCs derived from adipose tissue, bone marrow, placenta, umbilical cord, dental pulp, and tonsils have significant therapeutic potential in the treatment of skeletal diseases such as osteoporosis, osteogenesis imperfecta, osteoarthritis, and spinal cord injuries. The primary mechanisms of action include the modulation of the balance between osteogenesis and bone resorption, enhancement of chondrocyte proliferation, inhibition of angiogenesis, prevention of chondrocyte mineralization, exertion of paracrine signaling, facilitation of vascular and neuronal axon regeneration, and attenuation of ferroptosis. The figure was created with BioRender.com. NGF nerve growth factor, IL-1β interleukin-1 beta, MMP-13 matrix metalloproteinase-13, TNF-α tumor necrosis factor-alpha, ADAMTS-5 disintegrin and metalloproteinase with thrombospondin motifs 5, IL-10 interleukin-10, Col2 type II collagen, SOX9 SRY-box transcription factor 9, β-NGF beta-nerve growth factor, PI3K/AKT/mTOR phosphoinositide 3-kinase/protein kinase B/mammalian target of rapamycin, BDNF brain-derived neurotrophic factor, PTGS2 prostaglandin-endoperoxide synthase 2 and ROS reactive oxygen species

Significant strides have been made in evaluating the therapeutic efficacy of MSCs sourced from diverse origins in various skeletal pathologies (Fig. 4). MSCs obtained from adipose tissue, bone marrow, placenta, umbilical cord, dental pulp, and even tonsillar tissue have demonstrated encouraging therapeutic outcomes in the management of conditions such as osteoporosis, osteogenesis imperfecta, spinal cord injuries, and osteoarthritis.

### Osteoporosis

Osteoporosis is an age-related bone disease characterized by reduced bone mass and alterations in bone microstructure, leading to increased fragility and a greater risk of fractures, particularly among elderly individuals. The pathophysiology of osteoporosis involves a complex interplay of factors that contribute to bone loss, including age, mechanical stimuli, and hormonal changes. A significant factor in hormonal changes is the decrease in estrogen levels during menopause, which markedly accelerates bone remodeling and decreases bone density.^228^

MSCs are vital components of the bone marrow microenvironment owing to their multipotent capabilities, which allow them to differentiate into various cell types, including osteoblasts, adipocytes, and chondrocytes.^229^ In osteoporosis, MSCs tend to favor adipogenic differentiation, which reduces bone formation and increases fat accumulation within the bone marrow, thereby exacerbating bone loss and increasing the risk of fractures.^230^ However, the inherent plasticity and regenerative potential of MSCs present promising therapeutic prospects for managing osteoporosis.^230^ By strategically modulating the differentiation pathways of BM-MSCs to promote osteogenesis rather than adipogenesis, innovative treatments can be developed to increase bone density and strength, offering a novel and practical approach to address this debilitating condition.

Bilateral ovariectomy (OVX) is a widely recognized surgical procedure for establishing animal models that mimic osteoporosis caused by estrogen deficiency. Numerous studies have consistently shown that MSCs offer promising therapeutic outcomes in the treatment of OVX-induced osteoporosis. Specifically, various types of MSCs, such as BM-MSCs,^231^ DPSCs,^232^ and AD-MSCs,^233^ have been demonstrated to increase bone strength, increase bone mass, and improve the number of trabeculae, highlighting their therapeutic potential in countering the effects of OVX-induced osteoporosis. Among these, AD-MSCs have emerged as a particularly effective treatment for OVX-induced osteoporosis. Study has shown that osteogenesis-induced AD-MSCs stimulated osteogenesis and suppressed adipogenesis in OVX-induced osteoporotic BM-MSCs through the activation of the bone morphogenetic protein 2/bone morphogenetic protein receptor type IB signaling pathway.^234^ Human tonsil-derived MSCs (T-MSCs) have been shown to increase bone mass in the proximal tibia and femoral head and increase serum osteocalcin levels, indicating their efficacy in treating OVX-induced osteoporosis.^235^ Notably, after two consecutive injections, no signs of liver or kidney toxicity were observed over a three-month period.

Senile osteoporosis is a metabolic disease driven by aging, and genetically modified mice are commonly used to construct senescence-accelerated models. The transplantation of AD-MSCs into the bone marrow of osteoporotic mice, such as OVX-senescence-accelerated mouse prone 8 (SAMP8), has been shown to effectively restore BMD at various skeletal sites, including the knee, femur, and spine.^236^ Interestingly, mice receiving young AD-MSCs exhibited significantly greater bone regeneration than those receiving aged AD-MSCs did, suggesting that the aging process diminishes the therapeutic potential of AD-MSCs in osteoporosis treatment. In SAMP6 mice, tail vein injections of T-MSCs have been shown to sustain osteocalcin production and prevent the accumulation of bone marrow adipose tissue.^237^ Additionally, alternative injection methods, such as the intratibial injection of AD-MSCs into SAMP6 mice, have been demonstrated to modify tibial trabecular bone mass and increase the expression of osteogenic markers.^238^ In Sca-1^-/-^ mice, the tail vein injection of BM-MSCs increased the bone formation rate and turnover rate and prevented age-related osteoporosis.^239^ These findings underscore the importance of exploring various MSC sources and delivery methods for the treatment of senile osteoporosis, highlighting the potential of MSCs in enhancing bone mass and quality and the influence of age on the efficacy of stem cell therapies.

Secondary osteoporosis is a skeletal disorder frequently associated with glucocorticoid use and immunodeficiency diseases. In MRL/lpr mice, which exhibit IL-17-dependent hyperimmune conditions, the bone marrow environment leads to the destruction of BM-MSCs, thereby inhibiting osteoblast function and accelerating osteoclast induction. Systemic transplantation of human BM-MSCs and stem cells from exfoliated deciduous teeth into MRL/lpr mice has been shown to enhance the functionality of damaged BM-MSCs by inhibiting IL-17, thereby restoring the balance between osteoblasts and osteoclasts and maintaining normal bone metabolism, which alleviates secondary osteoporosis.^240^ In another model of secondary osteoporosis, systemic infusion of BM-MSCs into mice with dexamethasone-induced osteoporosis demonstrated that these cells can home to and inhabit the bone marrow for at least four weeks.^241^

MSCs play a significant therapeutic role in treating osteoporosis resulting from various causes. They not only reside in the bone marrow but also contribute to the enhancement of the bone marrow microenvironment, thereby promoting bone health through the regulation of osteoblasts, osteoclasts, and immune cells. However, there is currently no standardized cell type, administration route, or optimal cell dosage for MSC-based therapies for osteoporosis (Table 2). Extensive experimental studies should establish a consensus on the optimal intervention method.

### Osteogenesis imperfecta

Osteogenesis imperfecta (OI), also known as ‘brittle bone disease’, is a rare heterogeneous heritable skeletal dysphasia characterized by bone fragility, skeletal deformities, growth deficiency and other secondary connective tissue defects.^242,243^ The prevalence of OI is approximately 6 to 7 per 100,000 new births.^244^ OI is a collagen-related disorder caused by defects in genes whose protein products are either directly involved in collagen synthesis or indirectly involved in collagen protein folding, posttranslational modification, processing, and trafficking.^243^ As a result, defects in these genes inhibited bone mineralization and osteoblast differentiation and ultimately inhibited bone formation.

Although increasing evidence has revealed the pathophysiology of OI over the past decade, there is limited management and treatment for OI patients at different developmental stages. Genetic therapy to correct the underlying molecular defects and potential disease phenotype of OI is an emerging field in animal and preclinical studies.^245^ Moreover, MSCs have attracted great attention from scientists and clinicians in preclinical and clinical studies because of their differential capacity to differentiate into chondrocytes and bone cells, migratory ability to damage sites, and immunomodulatory effects.

Infant OI can be diagnosed readily through mid-trimester fetal anomaly scans and/or prenatal genetic diagnosis achieved through standard amniocentesis or fetal blood sampling, which opens up the possibility of offering prenatal treatment.^246^ Moreover, early treatment for infants with OI during the first year of life involves multidisciplinary management that ensures optimal care focused on skeletal development, cervical spine deformity, pain management and neurocognitive development.^247^ To achieve this goal, prenatal transplantation of MSCs could be a way to ameliorate the disease process during rapid skeletal development.^246^ In one animal experiment, human fetal blood-derived MSCs were injected intraperitoneally at E14 gestation in an OIM mouse model at a high dose of approximately 10^9^ cells/kg fetal weight.^248^ Transplantation was associated with increased bone strength, thickness, and length, as well as fewer bone fractures, in 4- to 12-week-old OIM mice.^248^ More detailed data have shown that donor cells from human MSCs are found in bone tissues than in other organs, where MSCs actively contribute to bone formation and remodeling, especially at the site of fracture healing.^248^ In clinical trials, Blanc and colleagues^249^ treated a female fetus diagnosed with severe OI type III and received prenatal transplantation with 6.5 × 10^6^ cells/kg allogeneic HLA-mismatched male fetal MSCs at 32 weeks of gestation. These allogeneic fetal MSCs successfully engraft and differentiate into the bone of OI patients at 9 months of age, which helps reduce the bone fracture rate and maintains normal psychomotor development during the first 2 years of life.^249^ Prenatal transplantation via the MSC approach enhances bone growth during fetal development and early in bone development.

However, a single transplantation is insufficient, as patients present a plateau growth velocity at some point. Thus, re-transplantation of MSCs is necessary and efficient to resume the normal growth trajectory for OI patients. Gotherstrom et al.^244^ reported an additional postnatal boosting of human fetal MSCs (hfMSCs) for Blanc’s patient mentioned earlier. At 8 years of age, this patient received re-transplantation with 2.8 × 10^6^ cells/kg hfMSCs from the same donor. Together with this clinical case, the same research team reported the second clinical case of both prenatal and postnatal transplantation of hfMSCs for another OI patient.^244^ The second patient with OI type IV was transplanted with 3 × 10^7^ cells/kg hfMSCs at 31 weeks of gestation and retransplanted with 1 × 10^7^ cells/kg hfMSCs at 19 months of age. In both cases, prenatal transplantation was safe for fetal development and highly effective in preventing new fractures during pregnancy or infancy.

Currently, there are no licensed treatments for children with OI.^250^ Children with OI commonly receive bisphosphonates to achieve anti-fracture efficacy.^250^ To achieve postnatal treatment with MSC therapy in OI, intravenous and intraosseous administration has been shown to have direct or indirect effects on increased cell engraftment and bone formation.^251^ In this case, the transitory nature and short-term effects of MSC therapy limited its clinical benefits in OI treatment; repeated MSC infusions have been applied in clinical trials. With increasing evidence showing the safety and efficacy of MSC treatment in OI patients, much attention has been given to encouraging large-scale clinical trials and deciphering the underlying molecular and cellular mechanism behind MSC transplantation for OI treatment. For example, the Boost Brittle Bones Before Birth (BOOSTB4) is a multicenter clinical research program under the European Union’s Horizon 2020 research and innovation program that investigates the safety and efficacy of stem cell transplantation treatment for severe OI patients, including both prenatal and postnatal patients. Moreover, studies have shown that EVs released from mouse BM-MSCs are able to stimulate chondrocyte proliferation in the growth plate and accelerate bone growth in an OI mouse model.^252^ In summary, considering the complexity of OI disease and its difficulty in current therapy, MSC treatment could be a realistic way to achieve clinical benefits in OI patients.

### Osteoarthritis

Osteoarthritis (OA) is a progressive, age-related joint condition that is particularly prevalent among elderly individuals, postmenopausal women, and individuals participating in intense physical activities. Although current interventions mainly aim to alleviate symptoms, their ability to repair joint damage is still limited. Recent studies have underscored the roles of MSCs in potential cartilage repair, reducing inflammation, and regulating the immune response in individuals with OA. MSCs are considered to have potential as cell therapies that release paracrine factors when administered locally or systemically. These cells secrete various mediators that can promote the native regeneration and multiplication of tissue stem cells, prevent cell death, or slow down cartilage degeneration, thereby assisting in the restoration and preservation of cartilage health.^253^

#### Cartilage regeneration

MSCs can promote cartilage formation and repair. In OA, MSCs move to injury sites from subchondral bone, becoming chondrocytes and osteoblasts for tissue repair.^254^ The mitochondria of MSCs are transferred to key joint cells and retained within the joint, balancing cellular redox, energy, and metabolic homeostasis in osteoarthritis chondrocytes without eliciting an inflammatory immune response, thereby alleviating joint cartilage degradation and maintaining cartilage integrity in OA.^255,256^

BM-MSCs represent the most frequently utilized therapeutic source of MSCs because of their benefits, which include convenient availability, swift cell growth, and sustained differentiation capacity. Prasadam et al.^257^ demonstrated that the coculture of BM-MSCs with chondrocytes increased the expression of cartilage regeneration markers, such as collagen type II (Col2), aggrecan, and SOX9. Other studies have found that BM-MSC-Exos alleviate OA progression by reducing chondrocyte ferroptosis, promoting cartilage repair, and enhancing extracellular matrix synthesis.^258,259^

In addition to BM-MSC-Exos, other MSC-Exos were also reported to be efficacious in enhancing cartilage regeneration and preventing osteoarthritis. Intra-articular injection of UC-MSC-Exos in OA rat models reduced the levels of cytokines (TNF-α, IL-1β, and IL-6) in the synovial fluid, decreased the expression of MMP-13 and ADAMTS-5 in chondrocytes stimulated by IL-1β, and increased the expression of Col2 in chondrocytes, thereby promoting cartilage regeneration.^260^ Furthermore, Exos derived from human synovial mesenchymal stem cells overexpressing miR-140-5p were reported to enhance cartilage tissue regeneration and prevent knee osteoarthritis in a rat model.^261^

#### Anti-inflammatory and immunomodulatory effects

In OA, the delicate balance between proinflammatory and anti-inflammatory cytokines is a pivotal aspect of disease pathogenesis. The immunomodulatory capacity of MSCs is particularly noteworthy, as they have the capacity to suppress the production of proinflammatory cytokines and stimulate the release of anti-inflammatory factors.^262,263^ This action shifts the cytokine environment toward one that promotes tissue repair. In animal models, post-MSC treatment decreased the expression of serum IL-1β, TNF-α, MMP-13, and ADAMTS-5.^262^ After MSC treatment, the levels of inflammatory biomarkers in OA are significantly reduced, whereas IL-10 and TGF-β1 levels are increased.^263^ MSC-based therapies enhance s-GAG synthesis impeded by IL-1β and suppress IL-1β-induced NO and MMP13 production. These effects are partially abrogated by inhibitors of adenosine receptor activation, AKT, ERK, and AMPK phosphorylation.^264^

TLR4 signaling plays a significant role in the pathogenesis of OA. The activation of NF-κB, a downstream effector in the TLR4 signaling cascade, is implicated in processes associated with OA.^265^ Yoon et al.^266^ investigated the interaction between the PUM1 protein and the 3ʹ-untranslated region (3ʹ-UTR) of the TLR4 gene, as well as its effect on NF-κB activity in MSCs. Their study revealed that gene therapy utilizing a lentiviral vector to deliver the mouse PUM1 gene could be effective in maintaining the integrity of articular cartilage in OA model mice.

In summary, MSCs offer a multifaceted approach to OA treatment by directly participating in cartilage repair and modulating the cytokine balance to suppress inflammation and promote anabolic processes. The capacity of MSCs to interact with the intricate cytokine network in OA is particularly noteworthy, suggesting that MSCs could be valuable tools for altering disease progression and alleviating related symptoms.

#### Analgesic effects of MSCs

MSCs have emerged as a therapeutic strategy for OA, offering potential pain relief through various molecular mechanisms and clinical applications. Copp et al.^267^ evaluated 15 randomized controlled clinical trials (RCTs) and 11 nonrandomized RCTs in which culture-expanded MSCs were used for the treatment of knee OA and reported the net positive effects of MSCs on mitigating pain and symptoms. Ai et al.^268^ reported that MSCs and MSC-EVs could reduce pain in OA via direct action on peripheral sensory neurons.

Research has confirmed several crucial molecules that are believed to play a role in the development of OA pain, potentially serving as specific targets for pain management in this disease. Pain alleviation is associated with the capacity of AD-MSCs to mitigate neuroinflammation in both the peripheral and central nervous systems (CNS). Secretome analysis revealed 101 immune factors involved in this process.^269^ Nerve growth factor (NGF), which is overexpressed in a mouse OA model, is a potential pain target in OA.^270^ Effective pain suppression in rodents with OA can be achieved through soluble TrkA,^270^ anti-NGF antibody therapy,^271^ and TrkA receptor inhibition.^272^ The analgesic mechanism of MSCs also involves the cyclooxygenase 2/PGE2 pathway, which has been implicated in the relief of OA pain.^273^ Furthermore, MSC-Exos carrying bioactive molecules from parental cells, including noncoding RNAs and proteins, have been demonstrated to significantly affect the modulation of various physiological behaviors of cells in the joint cavity, making them promising candidates for cell-free therapy for OA.^274^ MSC-Exos modified with TGF-β1 have been shown to alleviate cartilage damage and pain in OA by inhibiting angiogenesis and suppressing chondrocyte calcification and osteoclast activity.^275^ In OA rats, IL-4-overexpressing MSC spheroids demonstrated superior cartilage preservation and analgesic effects compared with standard naive mesenchymal stem cells.^276^

However, as we aggressively advance MSCs toward clinical application for patient care, it is imperative to remain vigilant about potential risks. For example, the follow-up duration of the current study was insufficient to assess the long-term biosafety of MSCs. Acquiring an adequate quantity of MSCs might necessitate multiple invasive procedures for patients. Moreover, as research progresses, the focus has been on determining the ideal source for MSC derivation and optimizing delivery methods to increase their therapeutic potential in OA. Therefore, there is an urgent need to develop effective agents that effectively use MSCs as seed cells while avoiding their potential risks for the treatment of OA cartilage degeneration.

### Spinal cord injury

Spinal cord injury (SCI) is a serious condition affecting approximately 15–40 people per million worldwide and has rapidly increased with the progress of modern society.^277^ Current treatments are limited to symptom management and rehabilitation. There is growing interest in regenerative medicine, such as stem cell therapy, for SCI. In 2024, Bydon et al.^278^ reported that 70% of patients treated with intrathecal delivery of autologous culture-expanded AD-MSCs for SCI experienced improvement compared with their pre-injection status.

SCI results from initial mechanical damage and subsequent secondary injuries, such as hemorrhage, ischemia, hypoxia, ionic imbalance, and free radical stress.^279^ Notably, oxidative stress resulting from ROS overproduction during the acute/subacute phase of secondary injury significantly reduces neuronal viability, hindering neural regeneration.^280^ MSCs have anti-inflammatory properties, promote vascular regeneration and the release of nutrients, and are a promising strategy for the treatment of SCI.

#### Anti-inflammation

MSCs play a pivotal role in the regeneration of SCI through their immunomodulatory capabilities, which are primarily mediated by cell contact and paracrine activity.^281^ In SCI, MSCs alleviate inflammation by targeting Exos to sites of inflammation, which in turn promotes tissue repair.^281^ A study by Liu et al.^282^ identified the TLR4/NF-κB/PI3K/AKT signaling cascade as a key mechanism in the modulation of microglial M1/M2 polarization by hypoxic exosomal miR-216a-5p. Additionally, the paracrine mechanism of BM-MSCs targets the TLR2/MyD88/NF-κB pathway in spinal cord dorsal horn microglia, leading to neuroprotection and sustained relief from neuropathic pain through the secretion of TSG-6.^283^ Transcriptome analyses revealed that IL-10 selectively enhances the migration and cytokine secretion of MSCs, which are essential for their anti-inflammatory therapeutic effects on SCI. These findings provide a robust basis for the future clinical application of genetically engineered MSCs.^284^ BM-MSCs are also known to inhibit the activation of p38 MAPK and Toll-like receptors, which are critical in SCI inflammation, thereby dampening the subsequent inflammatory response.^285^ The Notch pathway, which is important for neurogenesis and inflammation reduction post-SCI, is influenced by MSCs, indicating its role in modulating this pathway.^286,287^ The Nrf2 signaling pathway, which counters oxidative stress, is modulated by MSCs to reduce SCI-induced oxidative stress, with miR-200a targeting Keap1 to increase oxidative stress levels.^288,289^

#### Axon regeneration

The primary goal of post-SCI treatment is to repair damaged nerve cells and restore their function, which is essential for addressing the neuronal loss that leads to neurological deficits. MSCs play a significant role in this process. MSCs secrete neurotrophic factors, such as brain-derived neurotrophic factor (BDNF) and β-nerve growth factor (β-NGF), which are vital for neuronal survival and axon regeneration.^290^ Moreover, MSCs express neuronal markers such as β III tubulin, enolase 2, and microtubule-associated protein 1b (MAP1b) at the injury site, which are crucial for axon development. Transplantation of genetically engineered MSCs or MSC-laden biomaterials following SCI promotes axonal regeneration through enhanced trophic support and microenvironment modulation.^291,292^ Research has shown that CD271^+^CD56^+^ BM-MSC-Exo-mediated axon regeneration is significantly influenced by the miR-431-3p/RGMA axis.^293^ Guo et al.^294^ demonstrated that MSC-Exos loaded with phosphatase and tensin homolog small interfering RNA could reduce PTEN expression, thereby promoting axonal growth and neovascularization in the injured spinal cord region following intranasal administration. The Notch pathway, which governs neural stem cell proliferation and differentiation, can be inhibited by MSCs to promote neuron differentiation. Additionally, the Wnt/β-catenin pathway, which plays a role in neurodevelopment and survival, is activated by MSCs to increase axon regeneration post-SCI. The PI3K/AKT/mTOR pathway, known for its association with cellular growth and survival, is also activated by BM-MSCs to help restore motor function following SCI.^295,296^ BM-MSC-Exos have been shown to reduce inflammation and promote the differentiation of neural stem cells into neurons and oligodendrocytes. Another significant challenge in SCI is the formation of glial scars, which can hinder axonal regeneration. MSCs have demonstrated the ability to inhibit glial scar formation, thus fostering a more favorable environment for neural repair. This is achieved by reducing astrocyte activation and cytokine levels, particularly through the inhibition of the TGF-β/Smad signaling pathway, a key mediator of glial scarring.^297^ By addressing these challenges, MSCs represent a promising therapeutic approach for enhancing axon regeneration and improving outcomes in SCI treatment.

#### Vascular repair

Vascular injury following SCI can significantly exacerbate secondary SCI and result in neurological dysfunction. Strategies targeting angiogenesis have demonstrated the potential to enhance functional recovery post-SCI. A crucial role of UC-MSC-EVs was reported in the inhibition of DLL4 through the transfer of miR-27a-3p, which led to the promotion of angiogenesis and improved functional recovery after SCI.^298^ Huang et al.^299^ demonstrated that systemic administration of BM-MSC-Exos in the SCI rat model attenuated apoptosis and inflammation, promoted angiogenesis, and facilitated functional recovery after SCI.

#### Ferroptosis

Ferroptosis, a recently identified mechanism of iron-mediated oxidative cell death, is a significant contributor to neuronal loss following SCI. The transfer of mitochondria mediated by MSCs can replenish the neuronal mitochondrial population and suppress ferroptosis. This process occurs through mitochondrial fusion facilitated by intercellular tunneling nanotubes, which are crucial for counteracting the detrimental effects of ferroptosis on neurons.^300^ Laboratory studies have shown that MSC-Exos are capable of significantly reducing the generation of ferrous iron, malondialdehyde from lipid peroxidation, and ROS, as well as the expression of prostaglandin-endoperoxide synthase 2 (PTGS2), a factor that promotes ferroptosis.^301^ This reduction is mediated by the activation of the Nrf2/GCH1/BH4 pathway. Additionally, it has been reported that the MSC-exos lncGm36569 inhibited neuronal cell ferroptosis through the miR-5627-5p/FSP1 axis, thereby attenuating neuronal dysfunction.^302^ Despite these significant findings, how MSCs regulate ferroptosis and the specific regulatory pathways involved in the context of SCI are still being explored. The intricate mechanisms by which MSCs interact with and influence ferroptosis in SCI are an active frontier in scientific research, with potential implications for developing novel therapeutic strategies to protect against or repair the damage caused by SCI.

In summary, MSCs mainly repair the injured spinal cord through anti-inflammatory effects and promote nerve axon regeneration and vascular regeneration. MSCs offer advantages for the restoration of neurological function in individuals with SCI, but the precise mechanisms through which MSCs operate, as well as the cellular processes that hinder the reestablishment of neural circuits post-SCI, are still not fully understood.

## MSCs and cardiocerebral vascular diseases

### Stroke

**Fig. 5:**
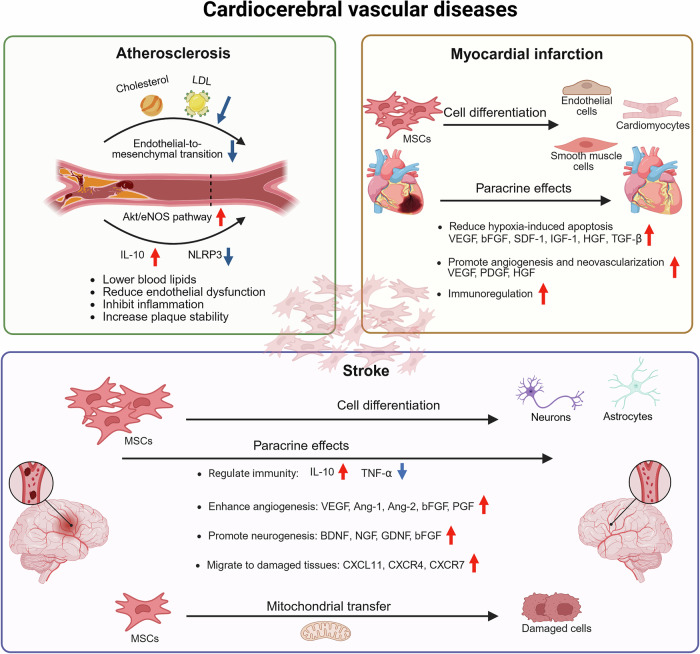
Mechanisms of MSC therapy for the treatment of cardiocerebral vascular diseases. MSCs derived from bone marrow, adipose tissue, the umbilical cord, and the gingiva possess therapeutic potential for the treatment of myocardial infarction, atherosclerosis, and stroke. MSCs exhibit the capacity to differentiate into neuronal, glial, cardiomyocyte-like, and vascular cell lineages, facilitating recovery from stroke and myocardial infarction. Furthermore, MSCs play pivotal roles in modulating immune responses, promoting angiogenesis, supporting neurogenesis, influencing cellular migration and homing, and mitigating hypoxia-induced apoptosis through paracrine signaling mechanisms. They also recover mitochondrial dysfunction by transferring healthy mitochondria to damaged cells by tunneling nanotubes, extracellular vesicles, gap junctions, and cell fusion. Additionally, MSCs contribute to the mitigation of atherosclerosis by lowering lipid levels, enhancing endothelial function, suppressing inflammatory processes, and promoting plaque stability. The figure was created with BioRender.com. VEGF vascular endothelial growth factor, bFGF basic fibroblast growth factor, SDF-1 stromal cell-derived factor-1, IGF-1 insulin-like growth factor-1, HGF hepatocyte growth factor, TGF-β transforming growth factor-beta, IL-6 interleukin-6, PGE2 prostaglandin E2, IL-10 interleukin-10, TNF-α tumor necrosis factor-alpha, Ang-1 angiopoietin-1, Ang-2 angiopoietin-2, BDNF brain-derived neurotrophic factor, NGF neurotrophic factor, GDNF glial cell line-derived neurotrophic factor, CXCL11 CXC chemokine ligand 11, CXCR4 CXC chemokine receptor 4, CXCR7 CXC chemokine receptor 7, PDGF platelet-derived growth factor, NLRP3 NOD-like receptor thermal protein domain associated protein 3

Stroke is the most common cerebrovascular disease and the second leading cause of death worldwide.^303,304^ MSCs, which have the potential to differentiate into a variety of cellular lineages and are noted for their relatively low risk of immune rejection in allogeneic transplants, have attracted the attention of researchers in the search for treatments for stroke.^305^ MSCs protect against stroke through multiple mechanisms, including mainly cell differentiation, paracrine effects, and mitochondrial transfer (Fig. 5).

#### Cell differentiation

One of the mechanisms by which MSCs exert their therapeutic effects is through direct cell replacement. BM-MSCs can differentiate into cells with neuronal characteristics after the application of trophic factors.^306^ Coculture of human UC-MSCs and activated astrocytes promoted neurotrophic factor secretion, which may facilitate both the neural differentiation of MSCs and endogenic neurogenesis.^307^

#### Paracrine effects

Although MSCs can differentiate into neurons and glial cells to repair structural damage, MSCs lack the voltage-gated ion channels expressed in functional neural cells to generate action potentials.^308^ Therefore, the paracrine effects of MSCs are likely the primary pathway through which MSCs confer therapeutic advantages in stroke management.^309^ The growth factors and cytokines released by MSCs can support stroke recovery through immune regulation, promotion of angiogenesis, and neuroprotection.

The immunomodulatory effects of MSCs are related to the regulation of proinflammatory and anti-inflammatory cytokines. Human BM-MSCs were cocultured with oxygen‒glucose deprivation–injured neurons, and MSCs exerted anti-inflammatory effects by secreting the anti-inflammatory factor IL-6 and decreasing the expression of the proinflammatory factor TNF-α.^308^ Transplanted BM-MSCs attenuated neurological injury after focal cerebral ischemia in rats by upregulating the expression of the anti-inflammatory cytokine IL-10 and downregulating the expression of the proinflammatory cytokine TNF-α.^310^ Importantly, MSCs can also play an immunomodulatory role through direct cell‒cell contact, whereas MSCs do not always exhibit immunosuppressive effects, which may be determined by local conditions in the microenvironment.^311^

After migrating to the infarcted area, MSCs secrete VEGF, angiopoietin-1 (Ang-1), angiopoietin-2 (Ang-2), basic fibroblast growth factor (bFGF), placental growth factor (PGF), etc., to increase angiogenesis in the ischemic core and border regions.^312–317^ MSCs achieve neuroprotection by directly releasing or increasing the release of endogenous neurotrophic factors, such as BDNF, NGF, GDNF and bFGF.^318–322^

In addition, MSCs have the ability to migrate to damaged tissues by releasing various chemokines/chemokine receptors.^323^ For example, the chemokine (C-X-C motif) ligand 11 (CXCL11) secreted by MSCs may interact with C-X-C motif chemokine receptor 3 (CXCR3) on human brain microvascular endothelial cells to induce the disassembly of tight junctions, which promotes MSC migration.^324^ CXCR4 and CXCR7 expressed by BM-MSCs jointly facilitate the migration of MSCs into the hippocampus of cerebral ischemia‒reperfusion rats.

#### Mitochondrial transfer

Mitochondrial transfer is a new mechanism of MSC therapy for diseases that has attracted wide interest. MSCs can target the recovery of mitochondrial dysfunction by transferring healthy mitochondria to affected cells through tunneling nanotubes, extracellular vesicles, gap junctions, and cell fusion.^181,325^ Human BM-MSCs can transfer healthy mitochondria to oxidation-damaged mouse primary neurons, thereby improving neuronal activity and neuron metabolism.^326^ Damaged brain microvascular host cells can accept mitochondria transferred by BM-MSCs, which significantly increases the mitochondrial activity of damaged microvasculature, promotes angiogenesis, reduces the size of cerebral infarction, and facilitates the functional recovery of stroke model rats.^327^

### Atherosclerosis

Atherosclerosis is a chronic inflammatory disease driven by endothelial dysfunction, lipid accumulation, and immune cell recruitment, ultimately leading to plaque formation within the intimal layer of the arterial wall.^328^ Multiple studies have shown that MSCs exhibit protective effects in atherosclerosis models induced by a high-fat diet in apolipoprotein E (ApoE)- or low-density lipoprotein receptor (LDLR)-knockout mice. The specific mechanisms by which MSCs affect the occurrence and progression of atherosclerosis include lowering blood lipids, reducing endothelial cell dysfunction, inhibiting inflammation, and increasing plaque stability^329^ (Fig. 5).

Hong et al.^330^ reported that administering G-MSCs to ApoE^-/-^ mice reduced total cholesterol and low-density lipoprotein levels while simultaneously increasing high-density lipoprotein levels. They reported a decrease in the expression of transcription factors involved in fatty acid biosynthesis and an increase in the expression of transcription factors regulating fatty acid β-oxidation in the mouse liver. Intravenous delivery of allogeneic BM-MSCs restored endothelium-dependent relaxation via an increase in phosphorylated Akt/eNOS in a mouse model of atherosclerosis.^331^ BM-MSC treatment attenuated palmitic acid-induced endothelial injuries by inhibiting endoplasmic reticulum stress and endothelial-to-mesenchymal transition.^332^

One study suggested that human G-MSC treatment alleviated atherosclerosis through decreasing monocytosis and modulating the activation and differentiation of macrophages in ApoE^−/−^ mice.^333^ Lin et al.^334^ found that BM-MSCs secreted IL-10, TGF-β, and PGE2 to promote the Treg differentiation by upregulating Foxp3 on the T lymphocyte surface, resulting in a favorable profile of immune components in plaques. BM-MSCs exert atheroprotective effects by increasing the number and function of Tregs and inhibiting macrophage foam cell formation.^335^

Human UC-MSC therapy improved plaque biomechanical properties by modulating NOD-like receptor thermal protein domain associated protein 3 (NLRP3) expression in plaques of ApoE^–/–^ mice, thereby increasing plaque stability and reducing the risk of plaque rupture.^336^

### Myocardial infarction

Myocardial ischemia (MI) is a severe cardiovascular condition that often leads to myocardial damage and decreased cardiac function. Similar to stroke, MSCs may play a role in the treatment of MI through cell differentiation and paracrine effects (Fig. 5).

#### Cell differentiation

Numerous studies have shown that MSCs can differentiate into cardiomyocyte-like cells both in vitro and in vivo.^337,338^ In addition, MSCs can also differentiate into vascular lineages (e.g., vascular smooth muscle cells and endothelial cells).^339,340^ AD-MSCs have the potential to differentiate into contractible vascular smooth muscle-like cells under costimulation with TGF-β and bone morphogenetic protein-4 (BMP4).^341^ Human BM-MSCs can differentiate into cells with endothelial cell phenotypic and functional characteristics in the presence of VEGF.^342^ Intravenously injected MSCs are capable of homing to the ischemic myocardium of rats, and the differentiation of MSCs into cardiomyocytes, smooth muscle cells, and endothelial cells can be observed from 1--4 weeks after transplantation.^343^

#### Paracrine effects

Although there is an enhancement in cardiac function following the transplantation of MSCs, evidence suggests that the engraftment and persistence of MSCs in areas of myocardial ischemia are neither substantial nor enduring. As a consequence, the functional benefits observed after MSC transplantation may be attributed to the release of soluble factors.^344^ MSCs can secrete VEGF, HGF, bFGF, IL-6, stromal cell-derived factor 1 (SDF-1), IGF-1, TGF-β, and PDGF for the treatment of MI.^345–347^ When cocultured with cardiomyocytes, BM-MSCs can release VEGF, bFGF, SDF-1, and IGF-1, which reduce hypoxia-induced apoptosis.^348^ Under hypoxia/reoxygenation conditions, human AD-MSCs upregulate the expression of several protective soluble factors (including HGF, VEGF-A, bFGF, and TGF-β) and inhibit the apoptosis of cardiomyocytes.^349^ Human BM-MSCs promote angiogenesis and neovascularization by secreting VEGF and PDGF to improve myocardial function and reduce scar size after MI in rats.^350^ Human UC-MSCs were found to secrete VEGF, HGF, and IL-6, which contribute to angiogenesis acceleration and immunoregulation in a porcine MI model.^351^ Furthermore, PGE2 secreted by MSCs plays a variety of roles in suppressing inflammation, such as inhibiting the polarization of M1 macrophages, the maturation and antigen presentation functions of DCs, and alleviating the inflammatory function of T cells.^352^

MSCs that have been modified by genetic engineering techniques to increase their functionality and efficacy in specific therapeutic applications are called gene-modified MSCs. Gene-modified MSCs can alleviate the massive loss of cells in the early stage of transplantation, improve the survival rate and ability of MSCs to target tissue, enhance paracrine effects and exert beneficial effects on MSCs to a greater extent.^353^ After treatment with HGF or VEGF gene-modified BM-MSCs, MI mice presented a smaller scar size, increased peri-infarct vessel density, and better preservation of left ventricular function.^354^ Compared with unmodified MSCs, VEGF gene-modified BM-MSCs are retained in greater numbers in the infarcted myocardium of rats and significantly reduce the number of apoptotic cells in the infarcted region.^355^ Another study also showed that the transplantation of VEGF gene-modified BM-MSCs stimulated extensive angiomyogenesis and improved left ventricular function in the infarcted hearts of rats.^356^ In summary, gene-modified MSCs could be a promising recent therapy for myocardial infarction and other diseases.

### Others

In other cardiovascular and cerebrovascular diseases, MSCs also show good efficacy. BM-MSCs were injected intravenously into rats with heart failure. These findings indicate that MSCs significantly reduce myocardial infarct size and interstitial fibrosis while improving heart rate variability.^357^ BM-MSC transplantation improved cardiac function in a rat model of dilated cardiomyopathy, possibly by inducing myogenesis and angiogenesis, as well as inhibiting myocardial fibrosis.^358^ Intramyocardial injection of BM-MSCs improved cardiac function in a rat model of furazolidone-induced dilated cardiomyopathy, probably by decreasing the ratio of type I/III collagen.^359^ In both chronic hypoxia and Sugen/hypoxia-induced pulmonary hypertension rat models, BM-MSCs decreased pulmonary arterial pressure, ameliorated collagen deposition, and reduced thickening and muscularization.^360^ Moreover, a growing body of research suggests that MSCs can treat traumatic brain injury by promoting neurological recovery and immunomodulation, inhibiting apoptosis, and promoting neurogenesis and angiogenesis.^361,362^

In summary, despite the encouraging results of many preclinical and clinical trials of MSC-based therapies for cardiocerebral vascular diseases (Table 2), many challenges remain to be overcome before MSCs can be widely used in clinical practice.

First, the optimal timing of intervention with MSCs remains controversial. Most studies have suggested that the transplantation of MSCs in the acute phase of stroke can result in improved therapeutic effects. Nevertheless, the role of multiple growth factors secreted by MSCs in promoting neurogenesis and neovascularization in the chronic phase of stroke should not be ignored. In addition, patients with postinfarction chronic ischemic heart disease and those with acute myocardial infarction may be treated with different therapeutic strategies.

Second, the culture time of autologous MSCs is long. Autologous MSCs are less suitable for the treatment of stroke, acute MI, traumatic brain injury, and other diseases associated with acute tissue injury.

Third, the best route of administration of MSCs and how to reach the lesion site effectively remain challenging. In the treatment of these diseases, various delivery methods, such as intravenous injection, ventricular injection, and myocardial injection, are used. Finding a suitable and efficient route of administration and improving the targeting of MSCs is the direction of our efforts.

Finally, patient comorbidities are also a challenge for MSC therapy. For example, many patients with stroke have comorbidities such as hypertension, diabetes, and heart disease. Antidiabetic drugs and antiplatelet drugs may affect the function of MSCs and limit their therapeutic effect.

## MSCs and neurodegenerative diseases

### Multiple sclerosis

Multiple sclerosis (MS) is a chronic autoimmune disease that affects the CNS, leading to demyelination and neurodegeneration. MSCs from different sources have shown potential for the treatment of MS.

Experimental allergic encephalomyelitis (EAE) mice treated with human BM-MSCs presented reduced EAE scores and reduced infiltration of inflammatory cells and demyelination in the spinal cord. The underlying mechanisms involve the downregulation of Th17 cells and the upregulation of Breg activity.^363^ Zhang et al.^364^ found that Exos from BM-MSCs ameliorated functional deficits via the enhancement of oligodendrocyte progenitor cell differentiation, remyelination, the modulation of microglial polarization, and a reduction in the inflammatory response in the CNS. BM-MSC transplantation ameliorated EAE by inhibiting the proliferation and activation of T cells, reducing the production of inflammatory cytokines, and regulating macrophage polarization.^365^ Bai et al.^366^ showed that HGF secreted by BM-MSCs ameliorated functional deficits, promoted oligodendrocyte and neuron development, and played a key role in myelin regeneration in EAE mice.

Liu et al.^367^ demonstrated that human UC-MSCs improved behavioral activity and reduced histopathological damage in EAE mice. In addition, tetramethylpyrazine enhanced the therapeutic effects of human UC-MSCs in EAE mice. Tetramethylpyrazine-stimulated human UC-MSCs significantly ameliorated MS, by attenuation of inflammation and demyelination.^368^ In a chronic MS model, intravenous administration of AD-MSCs and AD-MSC-EVs attenuated induced EAE by reducing T cell proliferation, mean clinical score, and demyelination.^369^ AD-MSCs increased the number of splenic Tregs and decreased IFN-γ secretion and cellular infiltration in the brain, thereby improving the severity of clinical scores in an EAE mouse model.^370^

In an EAE rodent model of MS, P-MSCs and P-MSC-EVs promoted myelin regeneration by inducing endogenous oligodendrocyte precursor cells to differentiate into mature myelinating oligodendrocytes.^371^ Intravenous injection of P-MSCs significantly ameliorated the MS course and reduced brain inflammation and neuronal degeneration in rats, which may be related to the release of BDNF, NGF, and neurotrophic factor 3 (NTF3) by P-MSCs.^372^

### Alzheimer’s disease

The pathological mechanisms underlying Alzheimer’s disease (AD) are complex and involve a cascade of neurodegenerative processes. One of the primary hypotheses is the amyloid cascade hypothesis, which suggests that the accumulation of beta-amyloid peptide (Aβ) triggers neuroinflammation, tau hyperphosphorylation, and ultimately neuronal death.^373,374^ MSCs function at different points in the pathologic process of AD.

#### Reducing Aβ deposition

In Aβ_1–42_-infused mice, the transplantation of P-MSCs, which are MSCs with anti-amyloidogenic and anti-neuroinflammatory effects, improved neuronal survival and neurogenesis and prevented memory deficiency.^375^ Overproduction of VEGF by BM-MSCs favored neovascularization and Aβ protein clearance, ultimately restoring memory and learning deficits in 2xTg-AD mice.^376^ Transplantation of human UC-MSCs into AD mice alleviated the rate of decline in memory and learning ability and improved the progression of disease in AD mice by reducing Aβ deposition.^377^

#### Inhibiting the phosphorylation of tau protein

UC-MSCs can repair damaged neuronal cells and significantly improve the learning memory and cognitive ability of AD model animals by regulating tau protein phosphorylation.^378^ BM-MSCs have a significant effect on the tau cell death cascade and can ameliorate the toxic effects of misfolded truncated tau.^379^ Multiple injections of BM-MSCs in young 3xTg-AD mice significantly decreased the pathological phosphorylation of tau at T205, S214, and T231.^380^

#### Anti-neuroinflammation

CNS damage due to abnormal accumulation of Aβ and tau proteins is the primary pathological mechanism of AD, and microglia-mediated immune injury is also involved in AD pathogenesis.^381^ AD-MSC transplantation ameliorated cognitive function in AD-induced rats, enhanced Aβ clearance, suppressed apoptosis, improved neurogenesis, and reduced neuroinflammation.^382^ By inducing a feed-forward loop involving alternative activation of microglial neuroinflammation, human UC-MSCs produce a sustained neuroprotective effect, thereby ameliorating disease pathophysiology and reversing the cognitive decline associated with Aβ deposition in AD model mice.^377^ In AD mice, injection of BM-MSC-EVs reduced plaque deposition and brain Aβ levels, decreased the activation of astrocytes and microglia, downregulated pro-inflammatory cytokines (TNF-α and IL-1β), and upregulated anti-inflammatory cytokines (IL-4 and IL-10).^383^ P-MSCs were reported to attenuate Aβ expression and improve cognitive impairment in an Aβ_1–42_-infused mouse model, possibly resulting from activated microglia.^375^

### Parkinson’s disease

Parkinson’s disease (PD) is a progressive neurodegenerative disorder characterized by the loss of dopaminergic neurons and the accumulation of insoluble cytoplasmic protein inclusions (Lewy bodies and Lewy neurons) in the substantia nigra compacta.

Various studies have indicated that MSCs derived from diverse sources can differentiate into dopaminergic neurons when subjected to specific combinations of growth factors and chemical agents (e.g., bFGF, BDNF, GDNF, IGF-1, retinoic acid, forskolin, ultrasound hedgehog, etc.).^384,385^ Offen et al.^386^ reported that transplanted BM-MSCs differentiated into dopaminergic-like cells and successfully improved the behavior of PD model mice.

Although differentiated MSCs exhibit neuronal morphology, express neuronal markers, and secrete dopamine, it has been suggested that they lack neuronal excitability.^387^ Moreover, numerous studies have shown that the conditioned medium of MSCs can exert an anti-PD effect, indicating that cell differentiation is not the most important mechanism by which MSCs treat this disease. MSCs secrete different growth factors, chemokines, inflammatory cytokines, and extracellular matrix components.^12^ A variety of growth factors, including VEGF, bFGF, IGF-1, and HGF, which are important effector molecules not only for promoting neovascularization and tissue repair but also for regulating the differentiation and function of MSCs, have been identified in the secretion profile of MSCs.^388^ Human BM-MSCs secrete IL-4 to polarize microglia toward an anti-inflammatory phenotype with enhanced phagocytosis to remove extracellular α-synuclein, suggesting a neuroprotective role in PD.^389^ Aizman et al.^390^ demonstrated that the extracellular matrix produced by BM-MSCs can support neuronal cell attachment and growth in vitro, which partly explains the benefit of transplanting BM-MSCs into neurodegenerative lesions of the CNS.

In addition to the aforementioned roles of BM-MSCs in PD, the transplantation of BM-MSCs reduced behavioral effects and partially restored the dopaminergic pathway in a rat model of PD.^391^ Human UC-MSCs were induced to transform into dopaminergic neurons in vitro, and transplantation of these cells into the striatum of rats with PD partially corrected injury-induced rotation.^392^ Three days after transplantation, subventricular neurogenesis was significantly increased in human AD-MSC-treated PD rats, which may be related to BDNF secreted by MSCs.^393^

### Amyotrophic lateral sclerosis

Amyotrophic lateral sclerosis (ALS) is a rapidly progressive and fatal neurodegenerative disease. ALS patients present with atrophy of the spinal cord, often with gray and white matter in the brain, and usually die within 3–5 years after the onset of clinical symptoms.^394^ BM-MSCs can improve functional motor outcomes and increase the lifespan and motor neuron count of ALS rats. Moreover, cells transplanted directly into the spinal cord survive until the end stage of the disease and are able to migrate into the white matter of the spinal cord.^395^ Uccelli et al.^396^ observed that systemic BM-MSC administration resulted in a decreased number of activated microglia and astrocytes as well as decreased expression of TNF-α and IL-1β in the spinal cord of ALS mice. Similarly, after the transplantation of human BM-MSCs into the lumbar spinal cord of ALS mice, the MSCs prevented the activation of astrocytes and microglia and delayed the decrease in the number of motor neurons associated with ALS, thereby improving motor function in these mice.^397^ Human BM-MSCs can increase the expression of the anti-inflammatory cytokine IL-10 in the lumbar spinal cord of ALS mice through paracrine effects, reduce astrocyte proliferation, regulate microglial activation, and delay motor neuron death and motor decline.^398^ Transplantation of human AD-MSCs in ALS mice provides neuroprotection through the production of growth factors (NGF, BDNF, IGF-1, and VEGF), delays disease progression, and prolongs the lifespan of ALS mice.^399^

### Huntington’s disease

**Fig. 6:**
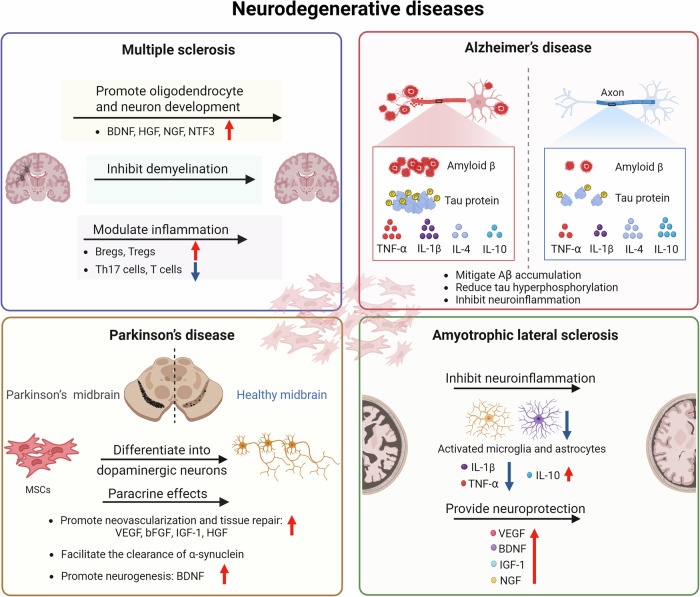
Mechanisms of MSC therapy for the treatment of neurodegenerative diseases. MSCs derived from the bone marrow, adipose tissue, umbilical cord, and placenta have therapeutic potential in the management of multiple sclerosis, Parkinson’s disease, amyotrophic lateral sclerosis, and Alzheimer’s disease. The primary mechanisms of action involve the enhancement of oligodendrocyte and neuronal differentiation, the inhibition of demyelination, and the attenuation of amyloid-beta accumulation and tau protein hyperphosphorylation. Additionally, MSCs exhibit paracrine activities, including the secretion of neuroprotective growth factors and the release of cytokines that modulate neuroinflammatory responses. The figure was created with BioRender.com. BDNF brain-derived neurotrophic factor, HGF hepatocyte growth factor, NGF nerve growth factor, NTF3 neurotrophin-3, IL-1β interleukin-1 beta, TNF-α tumor necrosis factor-alpha, IL-10 interleukin-10, VEGF vascular endothelial growth factor, IGF-1 insulin-like growth factor-1, bFGF basic fibroblast growth factor, Aβ amyloid-beta, and IFN-γ interferon-gamma

Huntington’s disease (HD) is an inherited neurodegenerative disorder caused by an abnormal amplification of the CAG repeats in the Huntington gene.^400^ The increased expression of BDNF, collagen type-I, and fibronectin in the brain after the transplantation of BM-MSCs into HD rats suggests that MSCs may treat HD through paracrine effects rather than neurological replacement.^401^ Overall, promising results have been obtained from preclinical studies regarding the therapeutic potential of MSCs in neurodegenerative diseases (Fig. 6). However, there are still many uncertainties and challenges facing the clinical efficacy of MSCs.

First, in the treatment of neurodegenerative diseases, how MSCs effectively cross the blood‒brain barrier is an inextricable problem. Intrathecal administration of MSCs has been shown to have better therapeutic effects on MS patients than intravenous administration.^402^ Intranasal administration is a noninvasive and rapid route of administration for the treatment of CNS disease. Preclinical models have demonstrated that MSCs can be successfully delivered to the brain via this route, and therapeutic outcomes have been observed in PD, MS, and AD.^403,404^

Furthermore, it is necessary to determine the culture conditions of MSCs. MSCs with optimal therapeutic properties were generated by considering culture time, donor age, storage time, thawing time, etc. A standardized MSC preparation protocol can effectively reduce the inconsistency between different studies.

In the end, as mentioned in the treatment of cardiocerebral vascular diseases, the optimal route of MSC injection may vary from disease to disease. In MS, a multifocal and systemic disease, intravenous injection provides a direct and safe way to modulate the peripheral immune response. However, the distribution of MSCs after transplantation needs to be more localized and precise in AD and PD.

## MSCs and diabetes

In 2021, there were 529 million people with diabetes globally, with an age-standardized prevalence of 6.1%. It is estimated that by 2050, there will be 1.31 billion people living with diabetes globally.^405^ MSCs are emerging as promising therapeutic options for diabetes,^406^ particularly for diabetes types 1 and 2, where immune destruction of insulin-producing beta cells in the pancreas leads to absolute or relative insulin deficiency. Exogenous insulin has been a longstanding treatment for type 1 diabetes and remains the standard of care. However, therapeutic intervention to reverse the disease state is challenging because β-cell function is often reduced to only 10–30% of normal at diagnosis. MSCs are multipotent cells that can differentiate into various cell types and secrete bioactive factors that support tissue repair, modulate immune responses, and reduce inflammation.^407,408^ These properties make MSCs highly relevant for the treatment of diabetes, such as functional degeneration, the loss of insulin-producing pancreatic β-cells, vascular complications, and problematic wound healing.

Type 1 diabetes (T1D) is a chronic metabolic disease caused by the autoimmune destruction of insulin-secreting pancreatic β-cells.^409–411^ The innate immune system is disrupted in T1D, resulting in the progressive destruction of pancreatic islet β-cells, which is mediated by the infiltration of CD8^+^ T cells, NK cells, and B cells, followed by the activation of DCs and macrophages. Insulin deficiency eventually leads to metabolic imbalance and even hyperglycemia.

In addition to traditional treatments, MSC transplantation is promising for the treatment of T1D because of its immunomodulation after systemic injection.^412–414^ By regulating cytokine patterns from proinflammatory to anti-inflammatory and increasing the number of Tregs in the peripheral blood, blood glucose was controlled in the intervention group after the injection of BM-MSCs.^412^

Moreover, other studies support the use of MSC transplantation in the treatment of type 2 diabetes (T2D).^415–418^ Chronic inflammatory responses and innate immune disturbances in islets and insulin-sensitive tissues lead to the onset of T2D. MSC treatment can ameliorate hyperglycemia by improving insulin resistance and promoting immunosuppression. After 12 months, 1 in 5 patients in the UC-MSC group achieved the primary endpoint (the percentage of patients with HbA1c levels <7.0% and a ≥ 50% reduction in daily insulin usage at 48 weeks).^415^

The healing of wounds in patients with diabetes is often hindered by infection, peripheral neurovascular diseases, hypoxia, neuropeptide conduction, and other factors, which increase patients’ medical expenditure and prolong the length of hospital stay. Diabetic ulcers (DUs) are among the most common complications of diabetes, affecting approximately 20% of diabetic patients.^419^ Despite advances in wound care, the United States Centers for Disease Control reported that DUs remain the leading cause of nontraumatic amputations.^420^ The complex interplay of both intrinsic and extrinsic factors influences the development of DUs. Intrinsic factors, such as neuropathy and peripheral vascular disease, contribute to the condition’s onset. In contrast, extrinsic factors—including wound infections, callus formation, and increased pressure at the ulcer site—significantly impact the healing process of DUs.^421^ Hence, there is a need to improve the care and treatment of DUs.

A promising therapeutic treatment involving MSCs has been widely explored in patients with diabetic UC. The immune-modulating effects of MSCs are mediated by the paracrine factors they secrete, including bFGF, insulin-like growth factor 1 (IGF-1), VEGF, epidermal growth factor (EGF), tissue inhibitor of metalloproteinase-1 (TIMP-1), progranulin, and BDNF.^344^ Growing evidence suggests that many therapeutic effects of MSCs may result primarily from the secretion of paracrine factors through EVs rather than direct cellular engraftment and response at the injury site.^422–425^ One study revealed that UC-MSC-Exos may enhance diabetic wound healing.^426^ Research has demonstrated that UC-MSC-Exos improve wound closure rates in diabetic rats by promoting endothelial cell proliferation, reducing the wound area, decreasing inflammation, and increasing collagen fiber formation.^426^ Additionally, UC-MSC-Exos increased the expression of anti-inflammatory markers (CD206) and angiogenesis markers (CD31 and VEGF) and reduced the levels of the inflammatory cytokine TNF−α.^426^ Hence, the development of MSC therapies that affect multiple disease pathways is increasingly important for patient care. However, research is needed to identify and characterize the specific factors secreted by BMSCs that mediate their regenerative effects. Moreover, understanding the regulatory networks that control this secretion will be critical to harnessing the full potential of BMSCs in clinical applications.

A key feature of type 2 diabetes mellitus (T2DM) is insulin resistance, which is often triggered by glucotoxicity and prolonged inflammation in adipose tissue. In murine macrophages, Exos originating from adipose-derived stem cells can stimulate the release of anti-inflammatory factors, thereby promoting metabolic homeostasis.^427^ Hence, MSCs may be a promising therapeutic modality to improve insulin sensitivity. Compared with control adipocytes, insulin-resistant adipocytes treated with 20 μg/mL Exos for 72 h presented significantly increased glucose uptake following insulin stimulation (*P* < 0.05). These findings suggest that MSC-Exo treatment can substantially increase insulin sensitivity in insulin-resistant human adipocytes.^428^

In conclusion, MSC transplantation represents an exciting approach for treating complex diseases through the delivery of combinatorial therapies. However, longitudinal studies are necessary to track the fate of transplanted BMSCs and their long-term effects on tissue regeneration. This includes understanding whether they differentiate, integrate, or senesce over time and how these processes affect overall healing outcomes.

## MSCs and cancer/tumorigenesis

### Tumor tropism

Cancer, characterized by abnormal cell proliferation with the potential to invade surrounding tissues and metastasize to distant sites, is widely recognized as an inflammatory disease. In the United States, approximately 1.7 million new cancer cases are diagnosed annually, with an average of more than 4,800 cases diagnosed daily.^429^ Conventional chemotherapy is effective against tumor cells but is often nonspecific, causing considerable side effects during cancer treatment.^430^ There is a need to develop precision medicine for tumor therapy.

MSCs exhibit a natural propensity for migration toward neoplastic sites, a phenomenon referred to as tumor tropism.^431^ This intrinsic characteristic makes MSCs highly promising candidates for the targeted delivery of therapeutics in oncological interventions.^432^ The mechanism underlying tumor tropism is thought to be mediated by interactions between specific receptors and chemokines, which promote the recruitment of ancillary cells to the tumor microenvironment. This recruitment process is influenced by various growth factors, including PDGF and IGF.^432^ However, one of the key concerns in the use of MSCs for cancer therapy is their proangiogenic potential, which could inadvertently support tumor growth. MSCs naturally secrete proangiogenic factors such as VEGF, FGF, and IL-6, which can enhance tumor vascularization and growth. While this stromal support is detrimental to unmodified MSCs, recent advancements in bioengineering have enabled the reprogramming of MSCs to mitigate these adverse effects. To overcome the protumorigenic limitations of MSCs, researchers have focused on genetic modifications that enable these cells to function as delivery systems for anticancer therapeutics. For example, certain miRNAs (e.g., miR-124) have been loaded into MSCs to suppress tumor growth by inhibiting oncogenic pathways.^433^ Additionally, MSCs can be engineered to secrete tumor necrosis factor-related apoptosis-inducing ligand (TRAIL) and other proapoptotic factors to induce cancer cell death.^434^

Studies have shown that intravenous injection results in the accumulation of hMSCs at metastatic melanoma,^435^ glioma,^436^ and colorectal tumor sites.^437^ For example, after hMSC injection into the femoral vein or common carotid artery in rats with glioblastoma, cells were detected in the tumor’s peripheral zone, a region of active angiogenesis. The percentage of hMSCs that localized to glioblastoma differed by injection site: 0.02% for the femoral vein, 0.1% for the common carotid artery, and 0.5% for the internal carotid artery.^438^ Notably, ipsilateral injections near the tumor were more efficient than intracardiac injections were, suggesting that closer proximity of the injection site to the tumor improved therapeutic outcomes. Clinical studies need to address the gaps in patient selection criteria, optimal delivery techniques (e.g., local injection vs systemic delivery), and the development of appropriate outcome measures. Research should also focus on overcoming the challenges related to the scalability and cost-effectiveness of MSC-based therapies.

### Immunomodulatory effects

The immune system primarily fulfills the roles of immune surveillance, defense, and regulation, thereby preserving the organism’s homeostasis. Dysregulation of immune functions, whether insufficient or excessive immune responses, can lead to various diseases. The immunomodulatory properties of MSCs are intricately linked to their interactions with innate and adaptive immunity. These effects are primarily mediated through direct cell‒cell contact with immune cells and the secretion of paracrine factors, including cytokines, chemokines, growth factors, and other modulatory agents.

DCs, macrophages, and NK cells play critical roles in the innate immune system, whose immune response is modulated by MSCs in numerous autoimmune diseases.^439–442^ Hence, MSCs have immunologically privileged properties, the ability to survive in an allogeneic environment, and the ability to migrate to damaged tissues and tumor sites. MSCs can inhibit DC differentiation, maturation, and antigen-presenting functions, thereby disrupting the secretion of proinflammatory cytokines. Under the influence of MSCs, DCs can reduce the production of proinflammatory cytokines. However, the tolerogenic DC phenotype induced by MSCs is unable to activate CD4^+^ T cells and effectively suppresses delayed-type hypersensitivity responses in vivo. Research has reported that DCs activated by MSCs are capable of promoting Treg generation, thereby modulating immune responses and preserving immune tolerance.^443^

The differentiation and phenotypic polarization of macrophages are influenced by their interactions with MSCs, which secrete factors such as PGE2, IDO, IL-10, and TGF-β.^444^ In a murine model of incision injury, researchers reported that MSCs migrate to the injury site, promoting the differentiation of macrophages into the M2 phenotype, which plays a crucial role in incision repair.^445^

A significant proportion of MSCs have been reported to suppress the proliferation, differentiation, and activation of NK cells.^446,447^ MSCs regulate the activity of NK cells primarily by modulating activating receptor signaling rather than by reducing NK cell inhibitory signaling. Furthermore, upon preconditioning with IFN-γ, MSCs exhibit downregulation of UL16 binding protein-3, while the expression of inhibitory MHC class I molecules is upregulated. This adjustment helps protect MSCs from being lysed by large amounts of IFN-γ and activated NK cells within the inflammatory microenvironment. Therefore, IFN-γ pretreatment enhances the ability of MSCs to withstand the cytotoxic effects of NK cells.^448^

### Angiogenesis

As described above, MSCs have a unique ability to home to sites of injury, inflammation, and tumor tissue. This tumor tropism has been exploited in cancer therapy, but it also means that MSCs can be attracted to the tumor microenvironment, where they may support tumor growth. MSCs can promote angiogenesis through the secretion of factors such as VEGF. Rapid maturation and expansion of the tumor vasculature are crucial for meeting the elevated demands for nutrients and oxygen at primary and metastatic tumor sites.^449^ Numerous studies suggest that MSCs contribute to tumor angiogenesis by differentiating into pericytes or endothelial-like cells. These differentiated MSCs subsequently secrete proangiogenic factors, trophic factors, and cytokines, thereby promoting the formation of new blood vessels in tumors.^450,451^

## Clinical research progress and limitations

MSCs have demonstrated considerable potential in clinical applications owing to their ability to differentiate into multiple cell types, immunomodulatory properties, and paracrine signaling capabilities. Various sources of MSCs and MSC-EVs, such as bone marrow, adipose tissue, the umbilical cord, and placental tissue, have been explored in clinical settings in recent years (Table 2). In the 2020 s, MSCs emerged as a central focus in the field of regenerative medicine, with significant advancements in the clinical implementation of MSC-based therapies.^68,452^ As of January 2025, more than 10 MSC-based drugs have received marketing approval in many countries (see Table 1). Nevertheless, challenges persist, including the need for standardized protocols, large-scale production, rigorous quality control, and the assurance of long-term safety for these treatments.

### Routes of administration, optimal dosage, and frequency

The route of administration plays a crucial role in influencing the biodistribution and therapeutic effectiveness of MSCs, which can differ on the basis of the MSC source and the targeted pathology (Table 2). Intravenous injection is frequently utilized for systemic conditions, leveraging the inherent ability of MSCs to migrate to injury sites. Nonetheless, there is a risk of MSCs becoming sequestered in the lungs due to the pulmonary first-pass effect, which diminishes their availability at the intended site of action.^453^ Intra-articular injection, often employed for osteoarthritis, effectively delivers MSCs directly to joint tissues.^454^ Both AD-MSCs and BM-MSCs have been utilized in this setting, with AD-MSCs potentially providing superior cartilage regeneration owing to their adipogenic lineage. Intrathecal and intracerebral injections are applied in neurological contexts, particularly with UC-MSCs, which exhibit increased secretion of neurotrophic factors. However, these techniques are invasive and entail greater procedural risks.^455^ Localized injection at the site of injury has been investigated for wound healing and soft tissue regeneration, frequently employing AD-MSCs because of their accessibility and high yield.^456^

Establishing the optimal dosage and frequency for MSC administration remains a complex challenge. The dosages administered in clinical trials demonstrate significant variability, ranging from 2 × 10^5^ to 1.5 × 10^7^ cells per kilogram of body weight. Additionally, other investigations reported dosages ranging from 1 × 10^7^ to 9 × 10^8^ cells, irrespective of body weight (Table 2). The discrepancies in cell viability and potency across various sources of MSCs further complicate efforts toward standardization. Elevated doses may heighten the risk of adverse effects, such as vascular occlusion or immune reactions, whereas insufficient doses may not achieve the intended therapeutic outcomes. The frequency of administration also varies, with some studies advocating for a single injection and others recommending multiple doses to maintain therapeutic efficacy (Table 2).

### Potential risks

The safety profile of MSCs derived from various sources is variable, yet they are predominantly regarded as favorable because of their low immunogenicity and minimal tumorigenic potential.^457^ The sustainability of MSC-based therapies is a significant concern, as the longevity and persistence of MSCs following transplantation are not thoroughly understood. Most research indicates that MSCs have a transient presence within the body, implying that their therapeutic effects are primarily mediated through paracrine signaling rather than long-term engraftment.^458^ This situation raises critical questions regarding the necessity for repeated treatments and the potential cumulative risks associated with multiple MSC infusions.

MSCs are a heterogeneous population of cells with varying properties and potentials. While some subsets exhibit greater regenerative potential, others may be less effective or even dysfunctional in specific contexts. There is a need to characterize the distinct subpopulations within MSCs better, including their unique roles in tissue repair and how these subsets interact within the stem cell niche. Understanding the heterogeneity of MSCs could help optimize their therapeutic use. Heterogeneity presents a considerable challenge across all sources of MSCs.^459^ MSCs constitute highly diverse populations that are influenced by various factors, including donor age, health status, and tissue origin. BM-MSCs typically exhibit diminished proliferation and differentiation capabilities as the donor age increases, which adversely affects their therapeutic efficacy. The therapeutic effectiveness of standardized MSC products is essential prior to the initiation of clinical trials.^460^ This necessity has driven researchers to devise enhanced in vitro potency assessment techniques that accurately correlate the critical quality attributes of MSC products with their therapeutic capabilities.^461,462^ Currently, the predominant method for determining MSC product potency involves assessing the in vitro inhibition of T-cell proliferation via the use of activated CD4^+^ T cells.^463^ In comparison with other markers of immune regulatory function, such as IDO expression or TNF-α receptor expression, this approach is deemed more indicative of efficacy, as it offers a direct measurement of biological activity. Despite the unique characteristics of each MSC product, the existing data remain inadequate to conclusively ascertain how variations in tissue sources, isolation techniques, and culture processes influence their therapeutic efficacy, in addition to valuable observational findings.

The quality of MSCs is another paramount concern, especially during in vitro expansion.^464^ Extended culture periods can result in cellular senescence, reduced differentiation potential, and alterations in surface marker expression. To ensure the integrity of the cells, rigorous quality control measures are essential, which include the implementation of potency assays and characterization of surface markers (e.g., CD73, CD90, and CD105) across different MSC sources. The diminished efficacy of MSCs following cryopreservation represents a significant challenge in the development of high-quality products.^465^ This clinical obstacle may be more effectively mitigated by refining the processing techniques of MSCs rather than merely altering their physical and functional characteristics. The majority of MSC therapies necessitate in vitro cell expansion, subsequent cryopreservation until needed, thawing of the preserved cells at the point of care, and their eventual administration to patients.^466^ The protocols for handling MSCs during thawing and administration exhibit considerable variability across different clinical trials, which could profoundly influence therapeutic outcomes post-administration. The procurement of tissue for autologous MSCs may present challenges, particularly when sourced from elderly donors, as both the quantity and functionality of MSCs may be compromised. To address the issues of functional decline or unpredictable regenerative outcomes in MSC therapy, it is imperative to clarify the underlying therapeutic mechanisms. Furthermore, to address the pathogenic concerns and ethical implications associated with MSC therapy, there is an urgent need for innovative treatment strategies that leverage advanced technologies to enhance MSC therapy in the future.

### In vivo tracking methods

In vivo monitoring of MSCs is crucial for elucidating their behavior and therapeutic mechanisms post-transplantation. The tracking methodologies exhibit variability in sensitivity and applicability across diverse MSC sources.

MSCs can be tagged with superparamagnetic iron oxide nanoparticles (SPIONs) for magnetic resonance imaging (MRI) visualization.^467^ However, this technique may not effectively distinguish between viable and nonviable cells. Positron emission computed tomography (PET) provides high sensitivity and can be used to monitor radiolabeled MSCs.^468^ Nonetheless, PET involves exposure to ionizing radiation. Bioluminescence imaging (BLI) is frequently employed in preclinical investigations of genetically modified MSCs. The BLI has demonstrated efficacy in animal models but faces limitations in clinical settings due to the requirement for genetic alterations.^469^ Fluorescence imaging employs fluorescent dyes or luciferase for MSC tracking.^458^ MSCs have been successfully monitored via this approach in preclinical studies, although challenges related to tissue penetration and dye toxicity persist. The advancement of multimodal imaging techniques that integrate various methods (e.g., MRI/PET) presents a promising strategy to increase tracking precision and yield comprehensive insights into MSC dynamics in vivo.

While the clinical potential of MSCs from various sources is undeniable, several challenges must be addressed to optimize their therapeutic use. The risks associated with safety and sustainability, the need for standardized routes and dosages, the variability in cell quality, and the limitations in current in vivo tracking techniques highlight the complexity of MSC-based therapies. The regulatory approval process for MSC therapies is still evolving, with the FDA and other regulatory bodies working to establish clear guidelines for the clinical application of MSC-based products. Continued research and innovation in these areas are crucial for the successful translation of MSC therapies into widespread clinical practice.

## Conclusion and perspective

MSCs have revolutionized the landscape of regenerative medicine, emerging as versatile therapeutic tools for diverse human diseases. Their unique biological properties—including multilineage differentiation potential, immunomodulatory capacity, and paracrine signaling via cytokines and growth factors—position them as a bridge between fundamental biology and clinical translation. Over the past decade, preclinical and clinical studies have demonstrated their efficacy in treating immune-inflammatory disorders, respiratory diseases, musculoskeletal defects, cardiocerebral vascular pathologies, neurodegenerative conditions, metabolic disorders, and neoplastic diseases. However, the journey from bench to bedside has revealed both triumphs and tribulations, necessitating a critical synthesis of current knowledge and a roadmap for future innovation.

At the molecular level, MSCs exert therapeutic effects through dynamic interactions with the host microenvironment. In immune-mediated diseases such as GVHD and RA, MSCs modulate T-cell polarization and suppress proinflammatory cytokines and Treg expansion. For respiratory disorders such as COVID-19 and ARDS, MSCs and MSC-EVs have shown promise in mitigating cytokine storms and repairing the alveolar epithelium. In osteoarticular diseases, MSCs synergize with biomaterial scaffolds to enhance cartilage regeneration, whereas in cardiovascular repair, their angiogenic potential improves cardiac function post-myocardial infarction. Neurodegenerative applications, particularly in Alzheimer’s disease and Parkinson’s disease, highlight the role of MSC-secreted BDNF and VEGF in neuronal survival and synaptic plasticity. These advances underscore the importance of disease-specific mechanisms to optimize MSC functionality. Systematic integration of proteomic, metabolomic, and EV profiling will elucidate the pleiotropic mechanisms underlying MSC efficacy.

Despite numerous registered clinical trials in the field of MSCs in treating human disease, critical challenges persist in translating MSC biology into standardized therapeutics. First, the inherent heterogeneity of MSC populations, which is influenced by tissue source, donor age, and culture conditions, creates significant batch-to-batch variability. Standardization protocols, such as CRISPR-Cas9 editing for uniform gene expression or bioreactor-based expansion systems, are being explored to address this issue. Single-cell RNA sequencing and artificial intelligence-driven predictive modeling, combined with blockchain-enabled traceability systems, may also address current reproducibility concerns in multicenter clinical trials. Second, the transient survival of transplanted MSCs in hostile microenvironments limits their durability. Strategies such as hypoxia preconditioning and genetic engineering to overexpress antiapoptotic genes may enhance engraftment. Future clinical applications will likely involve MSC-based combination therapies with biologics, small molecules, or physical modalities. The development of theranostic MSC hybrids—equipped with real-time biosensors for treatment monitoring—could further improve therapeutic regimens. Third, safety concerns, including potential profibrotic effects or unintended differentiation, demand rigorous long-term monitoring. Recent developments in primed or licensed MSCs—pretreatment with cytokines to increase immunosuppressive activity—offer a path toward safer, more predictable outcomes. Good manufacturing practice (GMP)-compliant production and adequate cost-effectiveness remain critical for the global accessibility of MSC therapy. Concurrently, harmonized international regulatory frameworks must evolve to address unique challenges in MSC product characterization, particularly regarding genomic stability after extended passaging.

In conclusion, as we stand at the crossroads of stem cell biology and translational medicine, MSCs embody both the promise and complexity of regenerative therapies. By embracing interdisciplinary innovation—spanning bioengineering, computational biology, and immunology—we can unlock their full potential. The ultimate goal is not merely to treat diseases but also to redefine healing itself, transforming MSCs from a “one-size-fits-all” remedy into a personalized, mechanism-driven solution. The coming decade will witness a paradigm shift from empirical MSC administration to rationally designed, digitally enabled cellular therapeutics. Success will depend on interdisciplinary collaboration across stem cell biologists, computational modelers, and regulatory scientists. By addressing current limitations in mechanistic understanding, manufacturing scalability, and clinical trial design, MSC-based interventions may transition from investigational agents to first-line therapies for a broad spectrum of disorders spanning multiple organ systems and disease categories with heterogeneous etiologies. Continuous innovation in this field not only promises to redefine modern medicine but also challenges our fundamental understanding of cellular plasticity and tissue regeneration.
